# Growth Regulation in Amphibian Pathogenic Chytrid Fungi by the Quorum Sensing Metabolite Tryptophol

**DOI:** 10.3389/fmicb.2018.03277

**Published:** 2019-01-08

**Authors:** Elin Verbrugghe, Connie Adriaensen, An Martel, Lynn Vanhaecke, Frank Pasmans

**Affiliations:** ^1^Department of Pathology, Bacteriology and Avian Diseases, Faculty of Veterinary Medicine, Ghent University, Merelbeke, Belgium; ^2^Laboratory of Chemical Analysis, Faculty of Veterinary Medicine, Department of Veterinary Public Health and Food Safety, Ghent University, Merelbeke, Belgium

**Keywords:** tryptophol, quorum sensing, chytrid, growth regulation, autostimulation

## Abstract

Amphibians face many threats leading to declines and extinctions, but the chytrid fungal skin pathogens *Batrachochytrium dendrobatidis* (*Bd*) and *Batrachochytrium salamandrivorans* (*Bsal*) have been identified as the causative factors leading to one of the greatest disease-driven losses of amphibian biodiversity worldwide. Infection may lead to different clinical outcomes, and lethal infections are commonly associated with unrestricted, exponential fungal growth in the amphibian epidermis. Mechanisms underpinning *Bd* and *Bsal* growth in the amphibian host are poorly understood. Here, we describe a quorum sensing mechanism that allows cell-to-cell communication by *Bd* and *Bsal* in order to regulate fungal densities and infection strategies. Addition of chytrid culture supernatant to chytrid cultures resulted in a concentration-dependent growth reduction and using dialysis, small metabolites were shown to be the causative factor. U-HPLC-MS/MS and *in vitro* growth tests identified the aromatic alcohol tryptophol as a key metabolite in regulating fungal growth. We determined tryptophol kinetics in both *Bd* and *Bsal* and confirmed the autostimulatory mode of action of this quorum sensing metabolite. Finally, we linked expression of genes that might be involved in tryptophol production, with *in vitro* and *in vivo* chytrid growth. Our results show that *Bd* and *Bsal* fungi use tryptophol to act as multicellular entities in order to regulate their growth.

## Introduction

Amphibian populations are declining worldwide and are threatened in their existence. One of the major causes of decline is the emerging infectious disease chytridiomycosis, a fungal skin disease that affects amphibian species across all three amphibian orders and which has driven over 200 amphibian species to extinction during the past decades (Daszak et al., 1999, 2003; Lips et al., 2006; Skerratt et al., 2007; Fisher et al., 2009; Lötters et al., 2009; Stegen et al., 2017). *Batrachochytrium dendrobatidis* (*B. dendrobatidis, Bd*) and the more recently discovered *B. salamandrivorans* (*Bsal*) (Martel et al., 2013, 2014), have been identified as the etiological agents of chytridiomycosis. *Bd* and *Bsal* are primitive fungi belonging to the phylum Chytridiomycota and the order Rhizophydiales (Van Rooij et al., 2015). The life stages of both fungi are characterized by a motile flagellated spore (zoospore) and a zoosporangium as a reproductive body. The zoospores are the infective stage of these fungi and they parasitize amphibian skin, causing disturbance of skin functioning and death in these animals (Berger et al., 1998; Voyles et al., 2007, 2009; Carver et al., 2010; Marcum et al., 2010; Brutyn et al., 2012). Recently a second type of encysted non-motile spores has been described in *Bsal*, which can persist without a host for a long time while remaining infectious (Stegen et al., 2017).

Host-chytrid dynamics vary between *Bd* and *Bsal* and among amphibian species, populations, and even individuals (Garner et al., 2009; Vredenburg et al., 2010; Martel et al., 2014). Despite the increasing scientific attention to chytridiomycosis, mechanisms that influence host characteristics and *Bd*/*Bsal* population densities still remain poorly understood. Quorum sensing (QS) is a mechanism of cell-to-cell communication that allows unicellular organisms to determine their population density, in order to regulate their population behavior, including growth (Albuquerque et al., 2013). It is a strategy of communication by signaling molecules used to coordinate the expression of determinants necessary to cause disease and has been described as crucial for the development and maintenance of acute and chronic infections by many pathogenic bacteria (Ng and Bassler, 2009; Parker and Sperandio, 2009; Möker et al., 2010; Kesarwani et al., 2011; Bandyopadhaya et al., 2012; Que et al., 2013). The ability to send, receive, and process information allows unicellular organisms to act as an entity in order to increase their survival chance in complex environments like a host. In fungi, QS research is still in its infancy and most of the work has been done in *Candida albicans* and *Saccharomyces cerevisiae*, both belonging to the phylum Ascomycota and the order of Saccharomycetales. The aromatic alcohols farnesol, tyrosol, phenylethanol and tryptophol are known QS molecules in these fungi, involved in among others metabolic activity, growth inhibition, and the transition between the filamentous and solitary yeast form (Chen et al., 2004; Mullard, 2009; Weber et al., 2010; Albuquerque and Casadevall, 2012). The exact mechanism of farnesol production remains poorly understood, but it is known that farnesol is synthesized from farnesyl pyrophosphate, a metabolic intermediate of the ergosterol biosynthesis (Hornby et al., 2003). Phenylethanol, tryptophol, and tyrosol are synthesized from the amino acids phenylalanine, tryptophan, and tyrosine, respectively, by chemical degradation through a three-step biochemical reaction comprising transamination, decarboxylation, and reduction (Ehrlich pathway). Aromatic aminotransferases I and II, encoded by the genes *ARO8* and *ARO9*, are involved in the transamination step, and the aromatic decarboxylase encoded by *ARO10* catalyzes the decarboxylation step (Hazelwood et al., 2008). In *Saccharomyces cerevisiae* the secretion of these aromatic alcohols is tightly controlled by cell density and a feedback autoregulatory circuit has been described for tryptophol (Chen and Fink, 2006). Tryptophol activates the transcription factor Aro80p and, consequently, the expression of the *ARO9* and *ARO10* transaminase and decarboxylase genes, which results in a positive feedback loop (Chen and Fink, 2006).

Until now, no research has been performed investigating whether chytrid fungi are able to communicate with each other and by doing so, regulate their population density. In the present study, we aimed at investigating whether *Bd* and *Bsal* use a mechanism of cell-to-cell communication. To this extent, we examined whether (1) QS molecules accumulate in conditioned medium (CM) during *Bd* and *Bsal* growth, (2) QS molecules accumulate in a concentration that is proportional to the population cell density, (3) QS molecules induce a coordinated response in the entire population and (4) the QS phenotype is reproduced when added to the culture exogenously (Albuquerque and Casadevall, 2012). Secondly, we aimed at correlating cell density and QS production to gene expression of *Bd* and *Bsal* and we screened chytrid-infected skin tissue of the common midwife toad *Alytes obstetricans* (belonging to the order Anura and the family Alytidae) to determine *in vivo* relevance of the QS system.

## Materials and Methods

### Collecting Conditioned Medium

Culture supernatant, further referred as CM was collected from a 5-day-old *Bd* (JEL423: highly virulent global panzootic lineage) or *Bsal* (AMFP13/1) culture grown at, respectively, 20°C and 15°C, containing both sporangia and zoospores (mid to late-exponential phase) (Supplementary Figure 1). The supernatant was collected from the flask and centrifuged at 3,000 rpm for 5 min to remove floating spores and sporangia, and the CM was stored at -20°C until further use. CM originating from cultures at different growth stages ranging from lag phase, early-late exponential phase, to stationary phase (*Bd*: varying from 1 to 14 days old and *Bsal*: varying from 5 to 8 days old) were screened for the presence of tryptophol, farnesol, tyrosol, and phenylethanol.

### Effect of CM on *Bd* Growth *in vitro*

*Bd* spores were collected from a full-grown culture containing mature sporangia. Once the zoospores were released, the medium containing the zoospores was collected and passed over a sterile mesh filter with pore size 10 μm (Pluristrainer, PluriSelect). The flow-through was used as the zoospore fraction (>90% purity). To achieve maximal zoospore adherence to the wells, *Bd* spores (5 × 10^5^ spores per well) were seeded in TGhL medium (1.6% tryptone, 0.4% gelatin hydrolysate and 0.2% lactose in H_2_O) supplemented with 50% H_2_O in 24-well plates and incubated for 3 h. After the spores had attached to the bottom of the wells, the medium was replaced by 1 ml control TGhL medium, H_2_O, CM or TGhL medium supplemented with different concentrations (20, 40, 60, or 80%) of H_2_O or CM. After 5 days, total *Bd* counts were determined using quantitative PCR (Boyle et al., 2004). Results represent the means of three independent experiments conducted in triplicate.

### Role of Cell Density in the Regulation of *Bd* Growth

We analyzed whether cell density is a determining factor that influences *Bd* growth. Therefore, we collected supernatants of 5-day-old *Bd* cultures grown at increasing density: (CM1) 1 ml of a mature *Bd* culture + 19 ml of TGhL broth; (CM2) 2 ml of a mature *Bd* culture + 18 ml of TGhL broth; (CM3) 3 ml of a mature *Bd* culture + 17 ml of TGhL broth; (CM4) 4 ml of a mature *Bd* culture + 16 ml of TGhL broth; and (CM5) 5 ml of a mature *Bd* culture + 15 ml of TGhL. *Bd* spores (5 × 10^5^ spores per well) were seeded in 24-well plates and after adhesion for 3 h, the spores were treated with TGhL broth supplemented with 40% of these different CM. As a control, TGhL medium was supplemented with 40% H_2_O. After 5 days, total *Bd* counts were determined using quantitative PCR (Boyle et al., 2004). Results represent the means of three independent experiments conducted in triplicate.

### Role of Small Molecules in the Regulation of *Bd* Growth

Quorum sensing is a process that involves the production of small molecules (metabolites). In order to determine whether small metabolites are involved in the process of *Bd* growth regulation, we dialyzed the CM against fresh TGhL using Float-A-Lyzer^®^ G2 systems (Spectrum labs) with a pore size of 100–500 Da and 500–1,000 Da. *Bd* spores (5 × 10^5^ spores per well) were seeded in 24-well plates and after adhesion for 3 h the spores were treated with TGhL broth supplemented with 40% of the dialyzed CM (500 Da and 1,000 Da). This was compared to spores treated with TGhL and TGhL supplemented with 40% H_2_O or 40% undialized CM. After 5 days, total *Bd* counts were determined using quantitative PCR (Boyle et al., 2004). Results represent the means of three independent experiments conducted in triplicate.

### Detection of QS Molecules Using Ultra-High Performance Liquid Chromatography (U-HPLC) Hyphenated to Tandem Mass Spectrometry (MS/MS)

#### Reagents and Chemicals

We screened for four known fungal QS metabolites [tryptophol (3-(2-hydroxyethyl)indole), E-E farnesol, tyrosol (2-(4-hydroxyphenylethanol)), 2-phenylethanol; Sigma-Aldrich]. Primary stock solutions of these metabolites and 2-methylindole (Sigma-Aldrich) as internal standard (ISTD) were prepared in methanol at a concentration of 1,000 ng/μl. Working solutions were prepared in 0.075% formic acid in water/methanol (75/25) or in extracted TGhL medium.

#### Instrumentation U-HPLC-MS/MS

The LC system consisted of a Thermo Scientific (San Jose, CA, United States) Accela U-HPLC pumping system, coupled with an Accela Autosampler and Degasser. Chromatographic separation was achieved by reversed phase chromatography and gradient elution. Separation of the components was carried out on an Acquity UPLC BEH C18 1,7 μm 2.1 × 100 mm column (Waters, Ireland). The mobile phase constituting of 0.075% formic acid in water/methanol (75/25), was pumped at a flow rate of 0.3 ml/min. Optimized separation was obtained using a linear gradient starting with a mixture of 75% 0.075% formic acid in water/methanol (75/25). After 0.5 min the amount of methanol was increased to 90% over a time period of 2.5 min and kept there for 1.5 min. Next, the amount of methanol was increased to 100% in 0.1 min and kept there for 1.30 min. Finally, the column was allowed to re-equilibrate for 2 min at initial conditions, this before each run. All analytes could be separated in a total runtime of 8 min. Analysis was performed on a triple quadrupole mass analyzer (TSQ Vantage, Thermo Scientific, San Jose, CA, United States), fitted with an atmospheric pressure chemical ionization source operating simultaneously in positive and negative ion mode. The following working conditions were applied: spray voltage at 3.2 (+) and 3.2 (-) kV; vaporizer and capillary temperature at 140°C and 270°C, respectively; sheath and auxiliary gas at 20 and 2 arbitrary units, respectively; cycle time of 0.8 s. Argon pressure in the collision cell (Q2) was set at 1.5 mTorr and the mass resolution at the first (Q1) and third (Q3) quadrupole was set at 0.7 Da at full width at half maximum. Precursor ion, S-lens RF amplitude, and collision energy in Q2 were optimized individually per compound or transition (Table 1). Quantification and confirmation data were acquired in selected reaction monitoring (SRM) mode, and the transitions followed are displayed in Table 1. Instrument control and data processing were carried out in Xcalibur 2.0.7 SP1 (Thermo Scientific, San Jose, CA, United States).

**Table 1:** Collected SRM transitions and compound-specific MS parameters.

Analyte	*t*_R_ (min)	Precursor ion *m/z* (Da) (polarity)	Product ions *m/z* (Da)	S-lens (RF amplitude) (V)	Collision energy (eV)
Tyrosol	1.63	121.2 (+)	103.1, 91.1, 77.1, 51.1	52	37, 20, 16, 14
Tryptophol	3.01	162.1 (+)	144.1, 143.1, 117.1, 115.1	56	32, 22, 23, 13
Phenylethanol	3.16	105.2 (+)	102.8, 79.2, 77.1, 50.3	61	56, 6, 16, 20
2-Methylindole (ISTD)	3.71	132.2 (+)	117.2, 90.2, 89.1, 63.2	86	50, 38, 31, 21
E-E farnesol	5.37	205.4 (+)	149.3, 93.2, 91.2, 77.1	57	36, 35, 22, 11


#### Sample Extraction and Clean-Up

TGhL and H_2_O-based samples were centrifuged at 13,000 rpm for 5 min and the supernatant was passed through a polyvinylidene fluoride membrane filter (0,22 μm × 13 mm diameter). Subsequently, the filtered samples were diluted in 0.075% formic acid in water/methanol (75/25) supplemented with the ISTD (=2-methylindole) at a final concentration of 2.0 ng/μl. TGhL-based samples were diluted 1:5, whereas H_2_O-based samples 1:2.

#### Quality Assurance

Identification of the QS molecules was based on their retention time relative to that of the ISTD and on the ion ratios of the product ions, carried out according to the criteria described in CD 2002/657/EC (Commission Decision, 2002). After identification, the tryptophol concentration was calculated by fitting its area ratio in a calibration curve, established by blank medium or water spiked with the ISTD at 2.0 ng/μl and tryptophol in the range of 0.1–1,000 pg/μl. Area ratios were determined by integration of the area of tryptophol under the specific SRM chromatograms in reference to the integrated area of the ISTD.

### Influence of Tryptophol on *Bd* and *Bsal* Growth

*Bd* (5 × 10^5^ spores per well) and *Bsal* (7,5 × 10^5^ spores per well) spores were seeded in 24-well plates and after adhesion for 3 h, the spores were treated with TGhL medium or TGhL medium supplemented with different concentration of tryptophol (10–500 μM). After 5 days, total *Bd* and *Bsal* counts were determined using quantitative PCR (Boyle et al., 2004; Blooi et al., 2013). Results represent the means of three independent experiments conducted in triplicate.

### Fungal Cell Density Dependence of Tryptophol Production

To test whether tryptophol is produced in a cell density-dependent way, *Bd* and *Bsal* spores were seeded in TGhL medium diluted 1:2 in H_2_O, at a concentration of 5 × 10^4^, 10^5^, and 5 × 10^5^ spores for *Bd* and additionally 10^6^ spores for *Bsal*. Every condition was tested in sixfold. At day 1, 3, and 5 post seeding, tryptophol was determined in the supernatant and in order to determine the tryptophol production per zoospore genomic equivalent (GE), *Bd* and *Bsal* counts were determined using quantitative PCR (Boyle et al., 2004; Blooi et al., 2013). Therefore, *Bd* and *Bsal* fungi were scraped from the bottom of the well using a 1 ml pipette tip, transferred to an Eppendorf tube together with the supernatant of the well and centrifuged for 5 min at 3,000 rpm. The pellet was resuspended in 100 μl of Prepman Ultra reagent (Applied Biosystems), heated for 10 min at 100°C, and the samples were allowed to cool to room temperature for 2 min. Subsequently, the tubes were centrifuged for 2 min at 13,000 rpm and 50 μl of the supernatants was transferred to a new Eppendorf tube. Finally, each sample was diluted 1:10 in H_2_O. Suspensions containing standardized numbers of *Bd* and *Bsal* zoospores were prepared as previously described (Boyle et al., 2004; Blooi et al., 2013) and 10-fold serial dilution series ranging from 1,000 to 0.01 GEs of zoospores per real-time PCR mixture were prepared for *Bd* and *Bsal*.

### Gene Expression That Might Be Involved in Tryptophol Secretion

Tryptophol secretion can be linked to the expression of *ARO8*, *ARO9*, and *ARO10*, as shown in *Saccharomyces cerevisiae* (Avbelj et al., 2015). We now screened the genomes of *Bd* and *Bsal* for possible gene candidates involved in tryptophol production (*ARO8*, *ARO9*, *ARO10*). Therefore, we performed a BLASTP 2.2.30+ search of *Bd* JEL423 and *Bsal* proteins (Bioproject PRJNA13653 and PRJNA311566) against 198 UniProt ARO8 listed proteins, identifying OON05735.1 for *Bsal* and OAJ43843.1 for *Bd* as significant hits to ARO8. When setting the e-value cutoff to e^-50^, BSLG_04451 (OON05735.1) and BDEG_27157 (variant OAJ43843.1) hit to 67 and 83 ARO8 proteins from the UniProt list, respectively (Supplementary Table 1). When considering the conserved domains using the NCBI’s Conserved Domains database, high similarity to ARO8 of *Saccharomyces cerevisiae* (Bioproject: PRJNA128, Accession: NP_011313) was observed with e-values of 4.17e^-97^ and 1.39e^-85^ for *Bd* and *Bsal* candidates, respectively (Supplementary Figure 2) (Altschul et al., 1997; Marchler-Bauer et al., 2017).

Using the RNeasy plant kit (Qiagen), total RNA was isolated from a 3-, 4-, 5-, and 7-day-old *Bd* and *Bsal* culture containing different life stages. We also extracted RNA from H_2_O-collected *Bd* and *Bsal* spores at a density of approximately 5 × 10^5^ spores/ml and 5 × 10^4^ spores/ml, respectively (further called “spores low”). Expression levels in spores reaching a high density were analyzed by extracting RNA from PmTG-collected *Bd* and *Bsal* spores at a density of approximately 10^7^ spores/ml and 10^6^ spores/ml, respectively (further called “spores high”). Finally, we extracted RNA from skin tissue from *Bd*-infected *Alytes obstetricans* (10 mg per animal) (Table 2). The samples were homogenized in RLT buffer by beat beating (3 rounds of 1.30 min at 30 Hz) using a tissuelyser II (Qiagen). The RNA concentration was measured by absorbance at 260 nm using a NanoDrop spectrophotometer, and the quality of the RNA samples was checked using an Experion RNA StdSens Analysis kit (Bio-Rad). Total RNA (1 μg) was reverse transcribed to cDNA with the iScript cDNA synthesis kit (Bio-Rad). Expression levels were normalized to the expression of the three most stable reference genes (α-centractin, R6046, TEF1a or GAPDH), which were determined for each condition using geNorm (qBase, Biogazelle). The list of genes and sequences of the primers used for quantitative PCR analysis can be found in Table 3. Real-time quantitative PCR reactions were run in duplicate (technical replicate) and the reactions were performed in 10 μl volumes using the iQ SYBR Green Supermix (Bio-Rad) and 1 μl 1/5 diluted cDNA. The experimental protocol for PCR (40 cycles) was performed on a CFX384^TM^ RT-qPCR System with a C1000 Thermal Cycler (Bio-Rad, Hercules, CA, United States). The results were analyzed using the Bio-Rad CFX manager 3.1. Quantification cycle (Cq) values were obtained using auto baseline settings and they were applied per primer set. The threshold for maximum Cq difference between the technical replicates was set to 0.5. The results are shown as fold changes of mRNA expression of at least three biological replicates, which were calculated based on the CNRQ values obtained in qBase. Statistics were also performed on the CNRQ values.

**Table 2:** Overview of the skin samples used for the screening of *BDEG_27157* expression.

Amphibian host	Real-time PCR	Fungal RNA detection
**Species**		
*Alytes obstetricans*	20 days p.i.: 3500 GE per PCR reaction	Yes
*Alytes obstetricans*	41 days p.i.: 3260 GE per PCR reaction	Yes
*Alytes obstetricans*	24 days p.i.: 13200 GE per PCR reaction	Yes
*Alytes obstetricans*	65 days p.i.: 2940 GE per PCR reaction	Yes
*Alytes obstetricans*	20 days p.i.: 874 GE per PCR reaction	No
*Alytes obstetricans*	21 days p.i.: 4450 GE per PCR reaction	Yes


**Table 3:** List of genes and sequences of the primers used for quantitative PCR analysis.

Gene		Primers	Length (bp)	E (%)	*R^2^*	Slope	Melt T
*Bd:* α-centractin	F	GCAGCATGGAGTTGTCACTG					
	R	AGCTTGGTCACGATTGGAAC	Farrer et al., 2017				
*Bd:* R6046	F	GTCGTACTGGCAACCTCACC					
	R	ACATTGGGAGCAATCTCGAC					
*Bd:* TEF1a	F	CCTTCCCGTCCTACTGACAA					
	R	GAACAGTTCCGATTCCTCCA					
*Bd:* GAPDH	F	AAGCCTGCCAAGTACGAAGA					
	R	AAAGATGGAGCTGCGAGTGT					
*Bd:* BDEG_27157	F	GGCTCCAAAAGCAGGCATGT					
	R	TCGATCCCAGGAACAAGCAA	114	96,6	0,999	-3,408	81
*Bsal:* α-centractin	F	CCGGCTACCATTTTCATACG					
	R	CGATCGATGGGTAGCACTCT	Farrer et al., 2017				
*Bsal:* R6046	F	GTTGCCAAGTCTGCTGTGAA					
	R	ATCAAGCGAGGGTGCAGAC					
*Bsal:* TEF1a	F	TCCCACTGACAAACCTCTCC					
	R	CGACAGGTACTGTTCCAATACCAC					
*Bsal:* GAPDH	F	GCCAGCAAAATACGAGGAGA					
	R	CCATTCATGGGTCCATTAGC					
*Bsal:* BSLG_04451	F	GCTCGGATTGGTTGGGTCAG					
	R	TTGGATCCCCCATTTCTCCA	135	98,7	0,999	-3,353	82


### Autostimulatory Mode of Action of Tryptophol

A characteristic of tryptophol is that it has an autostimulatory mode of action. In *Saccharomyces cerevisiae* it has been shown that tryptophol activates the transcription factor Aro80p and, consequently, the expression of the *ARO9* and *ARO10* transaminase and decarboxylase genes, which results in a positive feedback loop (Chen and Fink, 2006). This implies that a high population density produces more tryptophol per cell than cells at a low density. To test whether this is also the case in chytrid cultures, *Bd* (5 × 10^5^ spores per well) and *Bsal* (7,5 × 10^5^ spores per well) spores were seeded in 24-well plates. After adhesion for 3 h, the spores were treated with control TGhL medium or tryptophol-supplemented medium (0,001–1 μM) and grown for 3 days at 20°C or 15°C. To determine the tryptophol background, we also included wells without spores, but with the respective control or tryptophol-supplemented media. The background tryptophol production was subtracted from the tryptophol production after 3 days. Subsequently, we determined the relative “extra” tryptophol production compared to the untreated control wells. Every condition was tested in triplicate for *Bd* and sixfold for *Bsal*.

### *In vivo* Experiments

The *in vivo* experiments were carried out in strict accordance with the recommendation in the European Convention for the Protection of Vertebrate Animals used for Experimental and other Scientific Purposes. The experimental setup and protocol was approved by the ethical committee of the Faculty of Veterinary Medicine (Ghent University 2016/120). All animals used were clinically healthy and free of *Bd* and *Bsal* as assessed by sampling the skin using cotton-tipped swabs and subsequent performing qPCR (Boyle et al., 2004; Blooi et al., 2013).

We analyzed whether *BDEG_27157* is expressed during the later stages of infection, when high chytrid densities are reached. Six captive bred midwife toads (*Alytes obstetricans*) were exposed to 1 ml of 10^6^ *Bd* spores per ml water for 24 h at 15 ± 1 °C. As a negative control we included seven animals that were sham treated with 1 ml water for 24 h. Animals were followed up by clinical examination and the infection load was followed up weekly by taking swabs on which we performed qPCR. The animals were euthanized if clinical symptoms were observed (e.g., lethargy, loss of appetite, or weight reduction). A part of the skin (10 mg) was stored in RNA later buffer for 24 h and subsequently stored at -70°C. Skin samples for histopathology were stored in formalin. Histopathology confirmed *Bd* infection in all inoculated midwife toads.

Secondly, we investigated whether tryptophol is produced on the skin of chytrid negative animals. Therefore, the placebo inoculum (*n* = 7) was recovered and we quantified tryptophol production using U-HPLC-MS/MS. In addition, this was also tested in alpine newts *(Ichthyosaurus alpestris* belonging to the order of Chordata and family of Salamandridae) (*n* = 4).

### Statistical Analysis

All statistical analyses were performed using SPSS version 25 (SPSS Inc., Chicago, IL, United States). Normality of the data was assessed using a Kolmogorov–Smirnov and Shapiro–Wilk test. If normally distributed data showed equal variances (Levene’s test), they were analyzed using an unpaired Student’s *t*-test or one-way ANOVA with a Bonferroni *post hoc* test to address the significance of difference between mean values with significance set at *p* ≤ 0.05. If equal variances were not assessed or if the data were not normally distributed, they were analyzed using the non-parametric Kruskal–Wallis analysis with significance set at *p* ≤ 0.05. Multiple comparisons were assessed by a Kruskal –Wallis analysis followed by pairwise Mann–Whitney *U*-tests, with a Bonferroni-corrected *p*-value (*p*-value = 0.05/number of comparisons) (Supplementary Table 2).

## Results

### Amphibian Chytrid Fungi Regulate Their Own Growth in a Cell Density-Dependent Manner Using Metabolites

**FIGURE 1:**
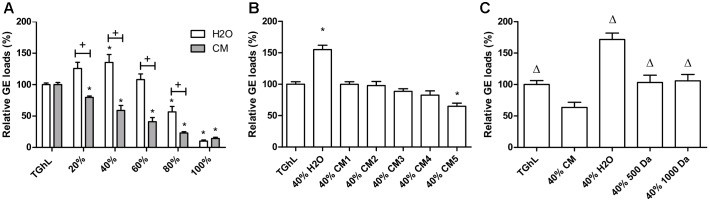
Effect of conditioned medium on *Bd* growth *in vitro*. Shown is the number of *Bd* cells grown for 5 days in TGhL medium supplemented with **(A)** different concentrations of conditioned medium (CM) or aqua dest (H_2_O), **(B)** 40% CM obtained from cultures with increasing density (CM1 to CM5) or aqua dest (H_2_O), **(C)** 40% CM, 40% dialyzed CM (500 Da and 1,000 Da) or 40% aqua dest (H_2_O). The GE numbers are expressed as percentage compared to the unsupplemented (TGhL) condition + standard error of the mean (SEM). **(A,B)** Statistical differences compared to the control (TGhL) are indicated by an asterisk, with **(A)** Bonferroni-adjusted *p*-value < 0.01 and **(B)** *p* < 0.05. **(A)** Significant changes compared to the H_2_O condition are depicted with +, with *p* < 0.05. **(C)** Significant differences compared to 40% CM treated spores are shown by Δ, with *p* < 0.05.

To evaluate the presence of QS regulation in chytrid, we added CM from a 5-day-old *Bd* culture to fresh spores and analyzed the possible effect on *Bd* growth after 5 days (Figure 1A). Supplementation of TGhL with CM resulted in a significant (*p* < 0.01) and dose-dependent decrease in *Bd* growth compared to spores grown in unsupplemented TGhL. In order to determine whether a decrease in available nutrients caused the drop in *Bd* growth, or whether the production of possible QS molecules was involved, we also included TGhL medium supplemented with H_2_O, showing no reduction in *Bd* growth.

Next, we prepared CM from *Bd* cultures with increasing density to analyze whether *Bd* regulates its growth in a cell density-dependent manner (Figure 1B). No significant effects were observed on *Bd* growth when supplementing its medium with CM derived from cultures with lower initial cell densities. CM originating from a dense culture did show a significant reducing effect (*p* < 0.05) in *Bd* growth, highlighting the cell density-dependent character of this inhibition mechanism.

Quorum sensing is a process that involves the production of small molecules (metabolites). In order to determine whether metabolites were involved in the process of *Bd* growth regulation, we analyzed the effect of CM following dialysis (500 and 1,000 Da) (Figure 1C). Supplementation with dialyzed CM did not cause a growth decrease, in contrast to a significant growth drop observed with undialyzed CM (*p* < 0.05). Both the CM originating from dialysis with a 500 and 1,000 Da filter reached growth levels similar to the control (unsupplemented TGhL), indicating that molecules with a molecular mass smaller than 500 Da are involved in the observed growth regulation by the CM.

### Tryptophol Is a Key Metabolite Involved in the Regulation of Chytrid Growth

**FIGURE 2:**
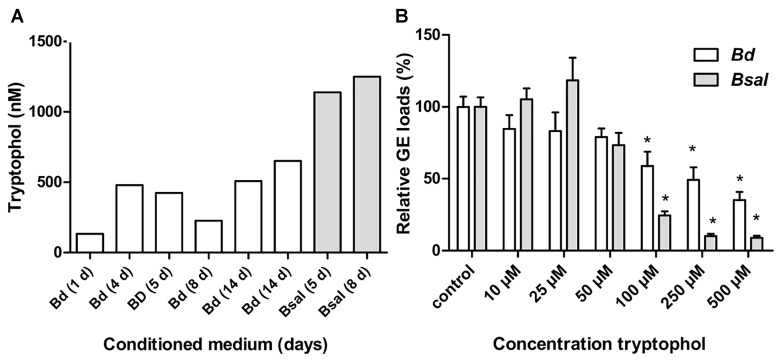
Tryptophol in conditioned media and its effect on chytrid growth. **(A)** Screening of different *Bd* (white bars) and *Bsal* (gray bars) conditioned media (varying from 1 to 14 days old) for tryptophol using U-HPLC-MS/MS. **(B)** Shown is the number of *Bd* (white bars) and *Bsal* (gray bars) cells grown in TGhL medium supplemented with different concentrations of tryptophol after 5 days. The GE numbers are expressed as percentage compared to the unsupplemented (TGhL) condition + SEM. Significant changes compared to control TGhL treated spores are shown by an asterisk, with a Bonferroni-adjusted *p*-value < 0.0083.

Conditioned medium obtained from both *Bd* and *Bsal* cultures at different growth stages (lag, exponential, and stationary phase) were screened for the presence of the known fungal QS molecules tryptophol, farnesol, tyrosol, and phenylethanol. To this extent, we utilized U-HPLC-MS/MS analysis to quantify the above-mentioned metabolites. In both the CM of *Bd* and *Bsal*, tryptophol was detected but farnesol, tyrosol or phenylethanol were not (Figure 2A). The production of tryptophol depends on the growth stage of the fungus, reaching concentrations between 133–652 nM for *Bd* and 1,139–1,251 nM for *Bsal*.

To test whether tryptophol indeed can regulate chytrid growth, we analyzed the direct effect of this metabolite on *Bd* and *Bsal* growth (Figure 2B). Tryptophol caused a dose dependent growth reduction in both *Bd* and *Bsal*, reaching significance at concentrations ≥100 μM (*p* < 0.0083).

**FIGURE 3:**
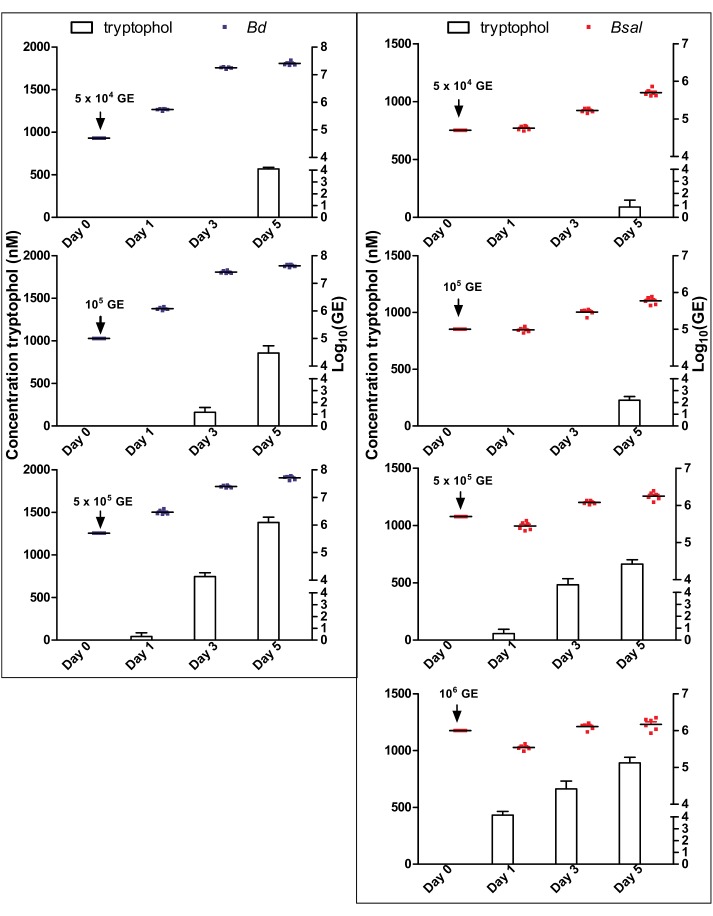
Correlation of *Bd* and *Bsal* growth to tryptophol production. Concentrations of *Bd* (left panel: blue dots) and *Bsal* (right panel: red dots) and production of tryptophol (histograms) over a growth period of 5 days at initial cell concentrations of 5 × 10^4^ GE, 10^5^ GE, 5 × 10^5^ GE for *Bd* and additionally 10^6^ GE for *Bsal*. Results represent the mean + SEM.

**FIGURE 4:**
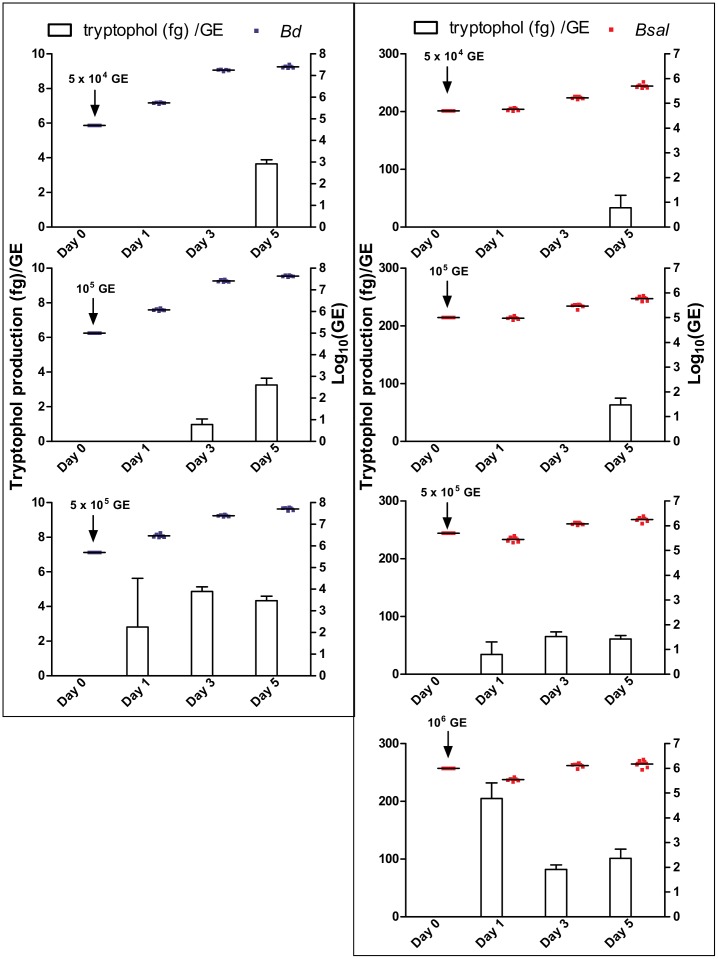
Tryptophol production per GE. Production rates of tryptophol (histograms; fg/GE) by *Bd* (left panel: blue dots) and *Bsal* (right panel: red dots) over a growth period of 5 days at initial cell concentrations of 5 × 10^4^ GE, 10^5^ GE, 5 × 10^5^ GE for *Bd* and additionally 10^6^ GE for *Bsal*. The production rates were calculated from the tryptophol and chytrid concentrations presented in Figure 3.

To determine the tryptophol kinetics, *Bd* and *Bsal* spores were seeded in 24-well plates at different concentrations and the tryptophol production was monitored at different days during its growth (Figure 3). Here, it can be observed that despite the difference in initial spore loads, the chytrid cultures reached similar densities. Although different initial starting concentrations were used, the GE loads after 5 days were very similar, with mean Log_10_(GE/ml) values of 7.4–7.7 for *Bd* and 5.7–6.2 for *Bsal*. It can also be seen that relevant tryptophol concentrations in the medium were detected when a certain concentration of viable cells was reached (i.e., the quorum was achieved), despite the differences in growth curve. For *Bd*, the critical point for tryptophol secretion was around 2.5 × 10^7^ GE/ml or a Log_10_(GE/ml) value of 7.4, whereas for *Bsal* at least a concentration of 3.5 × 10^5^ GE/ml or a Log_10_(GE/ml) value of 5.5 had to be reached. This indicates that in cultures with a higher initial start concentration of spores, tryptophol will be produced earlier in the growth process to regulate its own growth. For *Bsal* seeded at a density of 10^6^ GE/ml, even a small decrease in growth was observed after 1 day, corresponding with high production rates of tryptophol per GE (Figure 4).

### The Secretion of Tryptophol Is Subjected to Autostimulation

**FIGURE 5:**
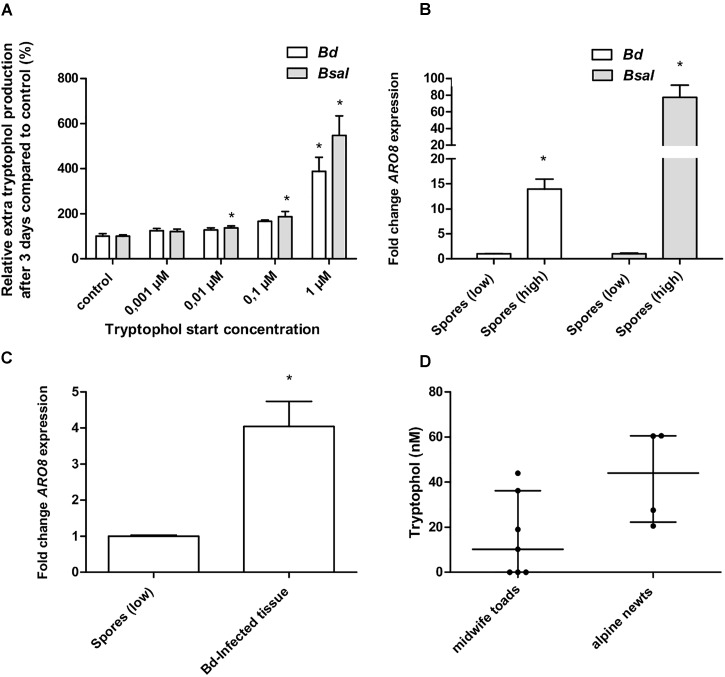
Autostimulation, *BDEG_27157*/*BSLG_04451* expression and *in vivo* tryptophol screening. **(A)** *Bd* and *Bsal* spores were seeded in wells treated with control TGhL medium or TGhL medium supplemented with different concentrations of tryptophol (0,001–1 μM). Using U-HPLC-MS/MS, the amount of tryptophol present in the wells after 3 days was determined. After subtracting the background tryptophol (tryptophol media without spores), we determined the relative “extra” tryptophol production compared to the control. Results represent the mean + SEM. An asterisk depicts a significant effect compared to the negative control, with a Bonferroni-adjusted *p*-value < 0.0125. **(B)** The data show the normalized *BDEG_27157* gene expression in *Bd* spores (10^7^ spores/ml) and *BSLG_04451* expression in *Bsal* spores (10^6^ spores/ml) originating from a PmTG-induced high-density culture (spores high), relative to H_2_O-induced spores at a density of 5 × 10^5^ *Bd* spores/ml and 5 × 10^4^ *Bsal* spores/ml (spores low), which is considered 1. **(C)** The data show the normalized *ARO8* gene expression of *Bd*-infected skin tissue of midwife toads (*n* = 5), relative to H_2_O-induced spores at a density of 5 × 10^5^ *Bd* spores/ml. **(B,C)** Results represent the mean + SEM. Significant differences compared to the control group (spores low) are indicated by an asterisk, with a *p*-value < 0.05. **(D)** Using U-HPLC-MS/MS, we determined the tryptophol concentration in skin washes, 24 h after contact with midwife toads (*n* = 7) and alpine newts (*n* = 4). Results are represented as the median with the interquartile range.

To determine whether tryptophol demonstrated an autostimulatory mode of action, as described for *Saccharomyces cerevisiae* (Chen and Fink, 2006), we analyzed if stimulation of *Bd* and *Bsal* with tryptophol results in an increased tryptophol production. The tryptophol production in 3-day-old cultures after tryptophol stimulation was determined, and we defined the “extra” tryptophol production by subtracting the background tryptophol (tryptophol media without spores). We then compared this to non-stimulated control cultures (Figure 5A). Autostimulation was observed in both *Bd* and *Bsal*. A concentration-dependent increase in “extra” tryptophol production was noticed, which reached significance (*p* < 0.0125) in both chytrid cultures challenged with 1 μM tryptophol. This indicates that cultures with high cell densities produced more tryptophol per cell than cells at a low density, confirming the results shown in Figure 4.

### *BDEG_27157* Is Expressed by Chytrids in Amphibians Colonized by High Chytrid Loads

We first optimized an RT-qPCR in *Bd* and *Bsal* targeting *BDEG_27157* and *BSLG_04451*, respectively. These targets hit to ARO8 (Supplementary Table 1 and Supplementary Figure 2), being aminotransferase I which is involved in the production of tryptophol (Hazelwood et al., 2008). In *Saccharomyces cerevisiae*, *ARO8* expression was shown to be directly linked to pathogen density and tryptophol concentrations produced by this organism (Avbelj et al., 2015). Tryptophol production in chytrid cultures depends on the density of the culture (Figures 3, 5A), suggesting that *BDEG_27157* and *BSLG_04451* expression will vary during chytrid growth and that *BDEG_27157* and *BSLG_04451* will be upregulated in high-density cultures. We now show that in both *Bd* and *Bsal*, these genes indeed are upregulated in high-density spore samples compared to low-density spore samples (*p* < 0.05) and that the expression differs depending on the growth stage of the chytrid culture (Figure 5B and Supplementary Figure 3).

By screening *Bd*-infected tissue of midwife toads for *BDEG_27157* expression, we analyzed whether tryptophol also plays a role during chytridiomycosis in amphibians, or whether its effect is limited to the *in vitro* situation. Results show that *BDEG_27157* was expressed in all the samples where fungal RNA was detected (five from six midwife toads) (Table 2). A significantly higher expression (*p* < 0.05) of this gene compared to H_2_O-induced *Bd* spores was observed, with an upregulation of 4.0 ± 0.70 (Figure 5C).

### *In vivo* Screening of Amphibian Skin Washes

We analyzed whether the U-HPLC-MS/MS method can be used as a screening method for tryptophol in skin washes (Figure 5D). Both in skin washes of midwife toads and alpine newts, tryptophol was found. In four out of seven midwife toads tryptophol was detected at levels ranging from 10.2 to 43.9 nM. In alpine newts (four out of four), slightly higher concentrations were observed, ranging from 20.6 to 60.6 nM.

## Discussion

Our study shows that the pathogenic chytrid fungi *Bd* and *Bsal* are capable of controlling their cell populations. Individual cells communicate with each other by secreting tryptophol in order to assess the population density and to coordinate their growth response. If a certain *Bd* or *Bsal* density is achieved, they start producing tryptophol with an autostimulatory mode of action, and when tryptophol reaches high concentrations in the exponential/stationary growth phase, this results in a growth reduction. It could be postulated that nutrient limitation occurs during these growth phases, leading to growth retardation. However, based on our experiments, we can conclude that this is not the case. Instead, we provide evidence for a cell density-dependent accumulation of tryptophol, resulting in a growth reduction proportional to the tryptophol concentrations reached in the conditioned media. By limiting their growth when reaching a certain density, these fungi prevent themselves from a quick death by the lack of nutrients and/or space. Instead, by actively diminishing their growth, they increase their chance of survival.

Besides growth regulation, QS molecules in fungi belonging to the phyla Ascomycota and Basidiomycota have been shown to regulate cell morphology, biofilm formation, resistance to oxidative stress and other processes (Albuquerque and Casadevall, 2012; Barriuso et al., 2018; Padder et al., 2018). Probably the influence of tryptophol on *Bd* and *Bsal* growth is only the tip of the iceberg. It is possible that if a certain tryptophol-threshold is reached, QS-dependent target genes are activated or repressed, leading to the regulation of the *Bd* and *Bsal* phenotype and genotype. Furthermore, QS is a process that can result in pathogen-host communication. Pathogen QS molecules can be received by host cells, or vice versa, host communication molecules can be received by the pathogen, consequently modulating host and pathogen behaviors and functions (Mullard, 2009). Some QS molecules can even be considered as virulence factors because of their effect on host cells (Winzer and Williams, 2001; Tateda et al., 2003). We showed that *BDEG_27157* is expressed during the later stages of chytridiomycosis, when high chytrid loads colonize the skin. This target shows homology to ARO8 in other organisms with e-values higher than e^-50^, hinting toward an ARO8 role. However, we were unable to perform a functional characterization of this gene. Creating a knockout mutant in *Bd* and *Bsal*, which has been done for *ARO8* in *Saccharomyces cerevisiae*, is currently impossible in chytrid research (Iraqui et al., 1998). Also, linking *BDEG_2715* and *BSLG_04451* expression to the conversion of tryptophan to tryptophol failed as we were unable to grow these fungi in minimal medium limiting amino acids availability. As such, whether tryptophol is produced by *Bd* and *Bsal in vivo* and its exact role remains to be elucidated.

A study by Loudon et al. (2014) showed that tryptophol can also be present on the skin of amphibians. They identified tryptophol in a co-culture of *Bacillus* sp. (phylum Firmicutes) and *Chitinophaga arvensicola* (phylum Bacteroidetes), skin bacteria from red-backed salamanders (*Plethodon cinereus*, belonging to the order of Chordata and family of Plethodontidae), as a potent inhibitor for *Bd*. Additionally, QS by tryptophol in other pathogens has been described (Gori et al., 2011; Albuquerque and Casadevall, 2012). These data indicate that the skin microbiota determines whether tryptophol is produced on an amphibian host, and at what concentrations. Tryptophol is recognized by both *Bd* and *Bsal*, indicating that cross-species communication can occur. Therefore, it is not unlikely that communication and interactions with other pathogens present on the skin occur. Depending on the skin microbiota this can lead to differences in disease progression. Amphibians could even use tryptophol as a defense mechanism against chytridiomycosis, which may be linked to inter- and intraspecific variation in chytrid sensitivity. We observed background levels of tryptophol on the skin of chytrid negative midwife toads and alpine newts, with individual differences. However, these concentrations were rather low and a lot lower than relevant levels produced by *Bd* and *Bsal* cultures, which is in line with the fact that these species are susceptible to chytrid infections (Bosch et al., 2001; Pasmans et al., 2010; Walker et al., 2010; Martel et al., 2014; Stegen et al., 2017). More research is needed to explore whether tryptophol production by skin bacteria is used as a defense mechanism, but screening skin washes of amphibians for tryptophol using the optimized U-HPLC-MS/MS method provides a valid non-invasive method for further research.

## Conclusion

In conclusion, we describe a QS mechanism in the pathogenic chytrid fungi *Bd* and *Bsal*. We identified tryptophol as a QS molecule with growth regulating capacities and described accumulation of this metabolite in conditioned media. We investigated the tryptophol kinetics during *Bd* and *Bsal* growth, and showed that its secretion is proportional to cell density. We provided evidence for autostimulation of tryptophol and our data suggest *in vivo* relevance for this QS metabolite during chytridiomycosis in amphibians. These results oppose the idea that *Bd* and *Bsal* fungi act as individual cells. Rather, *Bd* and *Bsal* are shown to be complex organisms that increase their survival changes by acting as one entity and detecting each other’s presence.

## Data Availability Statement

The raw data supporting the conclusions of this manuscript will be made available by the authors, without undue reservation, to any qualified researcher.

## Author Contributions

EV, CA, AM, and FP conceived the study, and participated in its design and coordination. EV and CA performed the experimental work. LV helped in the development and optimization of the U-HPLC-MS/MS method and the design and interpretation of the analysis results. EV wrote the first draft of the manuscript. All authors contributed to manuscript revision, read and approved the submitted version.

## Conflict of Interest Statement

The authors declare that the research was conducted in the absence of any commercial or financial relationships that could be construed as a potential conflict of interest.
