# Supplementary material for: Growth Regulation in Amphibian Pathogenic Chytrid Fungi by the Quorum Sensing Metabolite Tryptophol
Source: Front Microbiol. 2019 Jan 8;9:3277. doi: 10.3389/fmicb.2018.03277 (PMC6331427; doi:10.3389/fmicb.2018.03277)
Supplement: Supplementary file 1 [file Table_1.DOCX]

**Supplementary Table 1:** Results of a BLASTP 2.2.30+ search of *Bd* JEL423 and *Bsal* proteins (Bioproject PRJNA13653 and PRJNA311566) against 198 UniProt ARO8 listed proteins, identifying OON05735.1 for *Bsal* and OAJ43843.1 for *Bd* as significant hits to the ARO8. E-value cutoff was set to e^-50^.

**Query=** OON05735.1 hypothetical protein BSLG_04451, partial

[Batrachochytrium salamandrivorans]

Length=454

Score E

Sequences producing significant alignments: (Bits) Value

tr|M5E4G3|M5E4G3_MALS4 Similar to S.cerevisiae protein ARO8 (Ar...  [247](file:///C:\Users\Elin%20Verbrugghe\AppData\Local\Temp\Temp1_Re__Aro8_blasts.zip\Bsal_ncbi_aro_uniprot.html#BL_ORD_ID:104) 9e-78

tr|B0XTG0|B0XTG0_ASPFC Aromatic aminotransferase Aro8, putative...  [234](file:///C:\Users\Elin%20Verbrugghe\AppData\Local\Temp\Temp1_Re__Aro8_blasts.zip\Bsal_ncbi_aro_uniprot.html#BL_ORD_ID:45) 4e-72

tr|A1DHS0|A1DHS0_NEOFI Aromatic aminotransferase Aro8, putative...  [233](file:///C:\Users\Elin%20Verbrugghe\AppData\Local\Temp\Temp1_Re__Aro8_blasts.zip\Bsal_ncbi_aro_uniprot.html#BL_ORD_ID:118) 6e-72

tr|Q4X0F7|Q4X0F7_ASPFU Aromatic aminotransferase Aro8, putative...  [233](file:///C:\Users\Elin%20Verbrugghe\AppData\Local\Temp\Temp1_Re__Aro8_blasts.zip\Bsal_ncbi_aro_uniprot.html#BL_ORD_ID:52) 1e-71

tr|A0A2I1CNJ5|A0A2I1CNJ5_9EURO Putative aromatic aminotransfera...  [231](file:///C:\Users\Elin%20Verbrugghe\AppData\Local\Temp\Temp1_Re__Aro8_blasts.zip\Bsal_ncbi_aro_uniprot.html#BL_ORD_ID:134) 6e-71

tr|A1C7J1|A1C7J1_ASPCL Aromatic aminotransferase Aro8, putative...  [228](file:///C:\Users\Elin%20Verbrugghe\AppData\Local\Temp\Temp1_Re__Aro8_blasts.zip\Bsal_ncbi_aro_uniprot.html#BL_ORD_ID:34) 5e-70

tr|A0A0J5PYH6|A0A0J5PYH6_ASPFM Aromatic aminotransferase Aro8 O...  [222](file:///C:\Users\Elin%20Verbrugghe\AppData\Local\Temp\Temp1_Re__Aro8_blasts.zip\Bsal_ncbi_aro_uniprot.html#BL_ORD_ID:115) 1e-67

tr|A0A319EE16|A0A319EE16_9EURO Aromatic aminotransferase Aro8 O...  [221](file:///C:\Users\Elin%20Verbrugghe\AppData\Local\Temp\Temp1_Re__Aro8_blasts.zip\Bsal_ncbi_aro_uniprot.html#BL_ORD_ID:68) 2e-67

tr|A0A317WVZ6|A0A317WVZ6_9EURO Aromatic aminotransferase Aro8 O...  [221](file:///C:\Users\Elin%20Verbrugghe\AppData\Local\Temp\Temp1_Re__Aro8_blasts.zip\Bsal_ncbi_aro_uniprot.html#BL_ORD_ID:147) 2e-67

tr|A0A318ZNJ3|A0A318ZNJ3_9EURO Putative aromatic aminotransfera...  [221](file:///C:\Users\Elin%20Verbrugghe\AppData\Local\Temp\Temp1_Re__Aro8_blasts.zip\Bsal_ncbi_aro_uniprot.html#BL_ORD_ID:81) 2e-67

tr|B8MRR4|B8MRR4_TALSN Aromatic aminotransferase Aro8, putative...  [221](file:///C:\Users\Elin%20Verbrugghe\AppData\Local\Temp\Temp1_Re__Aro8_blasts.zip\Bsal_ncbi_aro_uniprot.html#BL_ORD_ID:151) 3e-67

tr|B8MRR5|B8MRR5_TALSN Aromatic aminotransferase Aro8, putative...  [218](file:///C:\Users\Elin%20Verbrugghe\AppData\Local\Temp\Temp1_Re__Aro8_blasts.zip\Bsal_ncbi_aro_uniprot.html#BL_ORD_ID:152) 9e-67

tr|V5FVN7|V5FVN7_BYSSN Aromatic aminotransferase Aro8, putative...  [219](file:///C:\Users\Elin%20Verbrugghe\AppData\Local\Temp\Temp1_Re__Aro8_blasts.zip\Bsal_ncbi_aro_uniprot.html#BL_ORD_ID:56) 2e-66

tr|B6Q744|B6Q744_TALMQ Aromatic aminotransferase Aro8, putative...  [218](file:///C:\Users\Elin%20Verbrugghe\AppData\Local\Temp\Temp1_Re__Aro8_blasts.zip\Bsal_ncbi_aro_uniprot.html#BL_ORD_ID:160) 5e-66

tr|A0A2G7FM37|A0A2G7FM37_9EURO Aromatic aminotransferase Aro8 O...  [217](file:///C:\Users\Elin%20Verbrugghe\AppData\Local\Temp\Temp1_Re__Aro8_blasts.zip\Bsal_ncbi_aro_uniprot.html#BL_ORD_ID:171) 7e-66

tr|A0A179FF75|A0A179FF75_METCM Aromatic aminotransferase Aro8 O...  [216](file:///C:\Users\Elin%20Verbrugghe\AppData\Local\Temp\Temp1_Re__Aro8_blasts.zip\Bsal_ncbi_aro_uniprot.html#BL_ORD_ID:142) 2e-65

tr|A0A1F7ZZH4|A0A1F7ZZH4_9EURO Aromatic aminotransferase Aro8 O...  [216](file:///C:\Users\Elin%20Verbrugghe\AppData\Local\Temp\Temp1_Re__Aro8_blasts.zip\Bsal_ncbi_aro_uniprot.html#BL_ORD_ID:70) 3e-65

tr|A0A2V5GVU1|A0A2V5GVU1_9EURO Aromatic aminotransferase Aro8 O...  [215](file:///C:\Users\Elin%20Verbrugghe\AppData\Local\Temp\Temp1_Re__Aro8_blasts.zip\Bsal_ncbi_aro_uniprot.html#BL_ORD_ID:43) 4e-65

tr|A0A317VFK7|A0A317VFK7_9EURO Aromatic aminotransferase Aro8 O...  [215](file:///C:\Users\Elin%20Verbrugghe\AppData\Local\Temp\Temp1_Re__Aro8_blasts.zip\Bsal_ncbi_aro_uniprot.html#BL_ORD_ID:143) 4e-65

tr|B8NHI9|B8NHI9_ASPFN Aromatic aminotransferase Aro8, putative...  [215](file:///C:\Users\Elin%20Verbrugghe\AppData\Local\Temp\Temp1_Re__Aro8_blasts.zip\Bsal_ncbi_aro_uniprot.html#BL_ORD_ID:154) 5e-65

tr|A0A2P2HCJ3|A0A2P2HCJ3_ASPFA Putative aromatic aminotransfera...  [215](file:///C:\Users\Elin%20Verbrugghe\AppData\Local\Temp\Temp1_Re__Aro8_blasts.zip\Bsal_ncbi_aro_uniprot.html#BL_ORD_ID:58) 5e-65

tr|A0A319D8H9|A0A319D8H9_9EURO Aromatic aminotransferase Aro8 O...  [214](file:///C:\Users\Elin%20Verbrugghe\AppData\Local\Temp\Temp1_Re__Aro8_blasts.zip\Bsal_ncbi_aro_uniprot.html#BL_ORD_ID:79) 9e-65

tr|A0A2V5IJE2|A0A2V5IJE2_9EURO Aromatic aminotransferase Aro8 O...  [214](file:///C:\Users\Elin%20Verbrugghe\AppData\Local\Temp\Temp1_Re__Aro8_blasts.zip\Bsal_ncbi_aro_uniprot.html#BL_ORD_ID:133) 2e-64

tr|A0A179HMM5|A0A179HMM5_9HYPO Aromatic aminotransferase Aro8 O...  [214](file:///C:\Users\Elin%20Verbrugghe\AppData\Local\Temp\Temp1_Re__Aro8_blasts.zip\Bsal_ncbi_aro_uniprot.html#BL_ORD_ID:102) 2e-64

tr|A0A2H3TBT2|A0A2H3TBT2_FUSOX Related to ARO8-aromatic amino a...  [211](file:///C:\Users\Elin%20Verbrugghe\AppData\Local\Temp\Temp1_Re__Aro8_blasts.zip\Bsal_ncbi_aro_uniprot.html#BL_ORD_ID:99) 5e-64

tr|A0A0L1JBH0|A0A0L1JBH0_ASPNO Putative aromatic aminotransfera...  [213](file:///C:\Users\Elin%20Verbrugghe\AppData\Local\Temp\Temp1_Re__Aro8_blasts.zip\Bsal_ncbi_aro_uniprot.html#BL_ORD_ID:114) 9e-64

tr|A0A1L7WCG1|A0A1L7WCG1_9HELO Related to ARO8-aromatic amino a...  [211](file:///C:\Users\Elin%20Verbrugghe\AppData\Local\Temp\Temp1_Re__Aro8_blasts.zip\Bsal_ncbi_aro_uniprot.html#BL_ORD_ID:136) 2e-63

tr|A0A1L7TM38|A0A1L7TM38_FUSMA Related to ARO8-aromatic amino a...  [209](file:///C:\Users\Elin%20Verbrugghe\AppData\Local\Temp\Temp1_Re__Aro8_blasts.zip\Bsal_ncbi_aro_uniprot.html#BL_ORD_ID:85) 2e-63

tr|A0A2H3SUE2|A0A2H3SUE2_FUSOX Related to ARO8-aromatic amino a...  [211](file:///C:\Users\Elin%20Verbrugghe\AppData\Local\Temp\Temp1_Re__Aro8_blasts.zip\Bsal_ncbi_aro_uniprot.html#BL_ORD_ID:97) 3e-63

tr|A0A1L7V7G8|A0A1L7V7G8_FUSPR Related to ARO8-aromatic amino a...  [210](file:///C:\Users\Elin%20Verbrugghe\AppData\Local\Temp\Temp1_Re__Aro8_blasts.zip\Bsal_ncbi_aro_uniprot.html#BL_ORD_ID:55) 5e-63

tr|A0A1L7S975|A0A1L7S975_FUSMA Related to ARO8-aromatic amino a...  [210](file:///C:\Users\Elin%20Verbrugghe\AppData\Local\Temp\Temp1_Re__Aro8_blasts.zip\Bsal_ncbi_aro_uniprot.html#BL_ORD_ID:84) 6e-63

tr|S0DIH8|S0DIH8_GIBF5 Related to ARO8-aromatic amino acid amin...  [210](file:///C:\Users\Elin%20Verbrugghe\AppData\Local\Temp\Temp1_Re__Aro8_blasts.zip\Bsal_ncbi_aro_uniprot.html#BL_ORD_ID:156) 8e-63

tr|A0A0I9YFI6|A0A0I9YFI6_GIBFU ARO8-aromatic amino acid aminotr...  [210](file:///C:\Users\Elin%20Verbrugghe\AppData\Local\Temp\Temp1_Re__Aro8_blasts.zip\Bsal_ncbi_aro_uniprot.html#BL_ORD_ID:89) 8e-63

tr|A0A0F0I3R4|A0A0F0I3R4_ASPPU ARO8 like protein OS=Aspergillus...  [209](file:///C:\Users\Elin%20Verbrugghe\AppData\Local\Temp\Temp1_Re__Aro8_blasts.zip\Bsal_ncbi_aro_uniprot.html#BL_ORD_ID:65) 8e-63

tr|A0A0S3G4K4|A0A0S3G4K4_BIOOC Aromatic acid aminotransferase O...  [209](file:///C:\Users\Elin%20Verbrugghe\AppData\Local\Temp\Temp1_Re__Aro8_blasts.zip\Bsal_ncbi_aro_uniprot.html#BL_ORD_ID:71) 3e-62

tr|A0A319BQ58|A0A319BQ58_9EURO Aromatic aminotransferase Aro8 (...  [208](file:///C:\Users\Elin%20Verbrugghe\AppData\Local\Temp\Temp1_Re__Aro8_blasts.zip\Bsal_ncbi_aro_uniprot.html#BL_ORD_ID:121) 3e-62

tr|A0A318YQ54|A0A318YQ54_9EURO Aromatic aminotransferase Aro8 O...  [207](file:///C:\Users\Elin%20Verbrugghe\AppData\Local\Temp\Temp1_Re__Aro8_blasts.zip\Bsal_ncbi_aro_uniprot.html#BL_ORD_ID:144) 5e-62

tr|A0A318Y4H9|A0A318Y4H9_9EURO Aromatic aminotransferase Aro8 O...  [206](file:///C:\Users\Elin%20Verbrugghe\AppData\Local\Temp\Temp1_Re__Aro8_blasts.zip\Bsal_ncbi_aro_uniprot.html#BL_ORD_ID:146) 6e-62

tr|A0A1L7W5V7|A0A1L7W5V7_FUSPR Related to ARO8-aromatic amino a...  [205](file:///C:\Users\Elin%20Verbrugghe\AppData\Local\Temp\Temp1_Re__Aro8_blasts.zip\Bsal_ncbi_aro_uniprot.html#BL_ORD_ID:53) 9e-62

tr|A0A319BKA8|A0A319BKA8_9EURO Aromatic aminotransferase Aro8 O...  [206](file:///C:\Users\Elin%20Verbrugghe\AppData\Local\Temp\Temp1_Re__Aro8_blasts.zip\Bsal_ncbi_aro_uniprot.html#BL_ORD_ID:119) 1e-61

tr|A0A1S9RYU0|A0A1S9RYU0_9EURO Putative aromatic aminotransfera...  [210](file:///C:\Users\Elin%20Verbrugghe\AppData\Local\Temp\Temp1_Re__Aro8_blasts.zip\Bsal_ncbi_aro_uniprot.html#BL_ORD_ID:42) 1e-61

tr|A0A100I5Z1|A0A100I5Z1_ASPNG Aromatic aminotransferase Aro8 O...  [204](file:///C:\Users\Elin%20Verbrugghe\AppData\Local\Temp\Temp1_Re__Aro8_blasts.zip\Bsal_ncbi_aro_uniprot.html#BL_ORD_ID:47) 2e-61

tr|A0A1B8GIZ3|A0A1B8GIZ3_9PEZI Aromatic/aminoadipate aminotrans...  [204](file:///C:\Users\Elin%20Verbrugghe\AppData\Local\Temp\Temp1_Re__Aro8_blasts.zip\Bsal_ncbi_aro_uniprot.html#BL_ORD_ID:153) 4e-61

tr|A0A2D3V0C5|A0A2D3V0C5_9PEZI Related to aromatic aminotransfe...  [203](file:///C:\Users\Elin%20Verbrugghe\AppData\Local\Temp\Temp1_Re__Aro8_blasts.zip\Bsal_ncbi_aro_uniprot.html#BL_ORD_ID:62) 7e-61

tr|A0A074XEX3|A0A074XEX3_AURPU Putative aromatic aminotransfera...  [199](file:///C:\Users\Elin%20Verbrugghe\AppData\Local\Temp\Temp1_Re__Aro8_blasts.zip\Bsal_ncbi_aro_uniprot.html#BL_ORD_ID:30) 3e-59

tr|G7XZZ6|G7XZZ6_ASPKW Aromatic aminotransferase Aro8 OS=Asperg...  [199](file:///C:\Users\Elin%20Verbrugghe\AppData\Local\Temp\Temp1_Re__Aro8_blasts.zip\Bsal_ncbi_aro_uniprot.html#BL_ORD_ID:67) 4e-59

tr|A0A1E1MDU4|A0A1E1MDU4_RHYSE Related to ARO8-aromatic amino a...  [199](file:///C:\Users\Elin%20Verbrugghe\AppData\Local\Temp\Temp1_Re__Aro8_blasts.zip\Bsal_ncbi_aro_uniprot.html#BL_ORD_ID:116) 5e-59

tr|A0A1E1KD92|A0A1E1KD92_9HELO Related to ARO8-aromatic amino a...  [199](file:///C:\Users\Elin%20Verbrugghe\AppData\Local\Temp\Temp1_Re__Aro8_blasts.zip\Bsal_ncbi_aro_uniprot.html#BL_ORD_ID:132) 5e-59

tr|A0A1E1KXI8|A0A1E1KXI8_9HELO Related to ARO8-aromatic amino a...  [198](file:///C:\Users\Elin%20Verbrugghe\AppData\Local\Temp\Temp1_Re__Aro8_blasts.zip\Bsal_ncbi_aro_uniprot.html#BL_ORD_ID:41) 8e-59

tr|A0A146F8N9|A0A146F8N9_9EURO Aromatic aminotransferase Aro8 O...  [194](file:///C:\Users\Elin%20Verbrugghe\AppData\Local\Temp\Temp1_Re__Aro8_blasts.zip\Bsal_ncbi_aro_uniprot.html#BL_ORD_ID:149) 5e-58

tr|A0A1T3CCB0|A0A1T3CCB0_9HYPO ARO8, Transcriptional regulator ...  [194](file:///C:\Users\Elin%20Verbrugghe\AppData\Local\Temp\Temp1_Re__Aro8_blasts.zip\Bsal_ncbi_aro_uniprot.html#BL_ORD_ID:145) 3e-57

tr|A0A0S3G4L3|A0A0S3G4L3_BIOOC Aromatic acid aminotransferase O...  [193](file:///C:\Users\Elin%20Verbrugghe\AppData\Local\Temp\Temp1_Re__Aro8_blasts.zip\Bsal_ncbi_aro_uniprot.html#BL_ORD_ID:36) 6e-57

tr|A0A167DEA0|A0A167DEA0_9ASCO Bifunctional 2-aminoadipate tran...  [192](file:///C:\Users\Elin%20Verbrugghe\AppData\Local\Temp\Temp1_Re__Aro8_blasts.zip\Bsal_ncbi_aro_uniprot.html#BL_ORD_ID:141) 1e-56

tr|A0A0D7BLG2|A0A0D7BLG2_9AGAR Aromatic aminotransferase Aro8 O...  [189](file:///C:\Users\Elin%20Verbrugghe\AppData\Local\Temp\Temp1_Re__Aro8_blasts.zip\Bsal_ncbi_aro_uniprot.html#BL_ORD_ID:50) 8e-56

tr|F2QQ55|F2QQ55_KOMPC Aromatic/aminoadipate aminotransferase 1...  [188](file:///C:\Users\Elin%20Verbrugghe\AppData\Local\Temp\Temp1_Re__Aro8_blasts.zip\Bsal_ncbi_aro_uniprot.html#BL_ORD_ID:35) 2e-55

tr|A0A1B2JEQ2|A0A1B2JEQ2_PICPA BA75_04174T0 OS=Komagataella pas...  [187](file:///C:\Users\Elin%20Verbrugghe\AppData\Local\Temp\Temp1_Re__Aro8_blasts.zip\Bsal_ncbi_aro_uniprot.html#BL_ORD_ID:131) 3e-55

tr|W0T517|W0T517_KLUMD Aromatic amino acid aminotransferase 1 O...  [184](file:///C:\Users\Elin%20Verbrugghe\AppData\Local\Temp\Temp1_Re__Aro8_blasts.zip\Bsal_ncbi_aro_uniprot.html#BL_ORD_ID:59) 6e-54

tr|V5IRM1|V5IRM1_NEUCR Aromatic aminotransferase Aro8, variant ...  [185](file:///C:\Users\Elin%20Verbrugghe\AppData\Local\Temp\Temp1_Re__Aro8_blasts.zip\Bsal_ncbi_aro_uniprot.html#BL_ORD_ID:93) 6e-54

tr|A0A319D396|A0A319D396_9EURO Aromatic aminotransferase Aro8 O...  [184](file:///C:\Users\Elin%20Verbrugghe\AppData\Local\Temp\Temp1_Re__Aro8_blasts.zip\Bsal_ncbi_aro_uniprot.html#BL_ORD_ID:130) 7e-54

tr|A0A1S7HCZ5|A0A1S7HCZ5_9SACH ARO8 (YGL202W) OS=Zygosaccharomy...  [183](file:///C:\Users\Elin%20Verbrugghe\AppData\Local\Temp\Temp1_Re__Aro8_blasts.zip\Bsal_ncbi_aro_uniprot.html#BL_ORD_ID:174) 1e-53

tr|A3LMX7|A3LMX7_PICST Aromatic amino acid aminotransferase OS=...  [182](file:///C:\Users\Elin%20Verbrugghe\AppData\Local\Temp\Temp1_Re__Aro8_blasts.zip\Bsal_ncbi_aro_uniprot.html#BL_ORD_ID:73) 4e-53

tr|A0A1Y2EGT2|A0A1Y2EGT2_9BASI Aromatic aminotransferase Aro8 O...  [182](file:///C:\Users\Elin%20Verbrugghe\AppData\Local\Temp\Temp1_Re__Aro8_blasts.zip\Bsal_ncbi_aro_uniprot.html#BL_ORD_ID:157) 8e-53

tr|A0A0H5BY90|A0A0H5BY90_CYBJA ARO8 protein OS=Cyberlindnera ja...  [180](file:///C:\Users\Elin%20Verbrugghe\AppData\Local\Temp\Temp1_Re__Aro8_blasts.zip\Bsal_ncbi_aro_uniprot.html#BL_ORD_ID:75) 1e-52

tr|H0GUJ4|H0GUJ4_SACCK Aro8p OS=Saccharomyces cerevisiae x Sacc...  [181](file:///C:\Users\Elin%20Verbrugghe\AppData\Local\Temp\Temp1_Re__Aro8_blasts.zip\Bsal_ncbi_aro_uniprot.html#BL_ORD_ID:48) 1e-52

tr|A0A017S5V8|A0A017S5V8_9EURO Putative aromatic aminotransfera...  [179](file:///C:\Users\Elin%20Verbrugghe\AppData\Local\Temp\Temp1_Re__Aro8_blasts.zip\Bsal_ncbi_aro_uniprot.html#BL_ORD_ID:128) 5e-52

tr|A0A100IGI9|A0A100IGI9_ASPNG Aromatic aminotransferase Aro8 O...  [177](file:///C:\Users\Elin%20Verbrugghe\AppData\Local\Temp\Temp1_Re__Aro8_blasts.zip\Bsal_ncbi_aro_uniprot.html#BL_ORD_ID:46) 3e-51

tr|A0A0J0XSH6|A0A0J0XSH6_9TREE Aromatic aminotransferase Aro8 O...  [176](file:///C:\Users\Elin%20Verbrugghe\AppData\Local\Temp\Temp1_Re__Aro8_blasts.zip\Bsal_ncbi_aro_uniprot.html#BL_ORD_ID:88) 6e-51

**Query=** OAJ43843.1 hypothetical protein BDEG_27157 [Batrachochytrium

dendrobatidis JEL423]

Length=470

Score E

Sequences producing significant alignments: (Bits) Value

tr|M5E4G3|M5E4G3_MALS4 Similar to S.cerevisiae protein ARO8 (Ar...  [270](file:///C:\Users\Elin%20Verbrugghe\AppData\Local\Temp\Temp1_Re__Aro8_blasts.zip\BD-jel_aro_uniprot.html#BL_ORD_ID:104) 2e-86

tr|A0A318ZNJ3|A0A318ZNJ3_9EURO Putative aromatic aminotransfera...  [231](file:///C:\Users\Elin%20Verbrugghe\AppData\Local\Temp\Temp1_Re__Aro8_blasts.zip\BD-jel_aro_uniprot.html#BL_ORD_ID:81) 7e-71

tr|A1DHS0|A1DHS0_NEOFI Aromatic aminotransferase Aro8, putative...  [229](file:///C:\Users\Elin%20Verbrugghe\AppData\Local\Temp\Temp1_Re__Aro8_blasts.zip\BD-jel_aro_uniprot.html#BL_ORD_ID:118) 4e-70

tr|A0A2I1CNJ5|A0A2I1CNJ5_9EURO Putative aromatic aminotransfera...  [227](file:///C:\Users\Elin%20Verbrugghe\AppData\Local\Temp\Temp1_Re__Aro8_blasts.zip\BD-jel_aro_uniprot.html#BL_ORD_ID:134) 2e-69

tr|B0XTG0|B0XTG0_ASPFC Aromatic aminotransferase Aro8, putative...  [226](file:///C:\Users\Elin%20Verbrugghe\AppData\Local\Temp\Temp1_Re__Aro8_blasts.zip\BD-jel_aro_uniprot.html#BL_ORD_ID:45) 3e-69

tr|Q4X0F7|Q4X0F7_ASPFU Aromatic aminotransferase Aro8, putative...  [226](file:///C:\Users\Elin%20Verbrugghe\AppData\Local\Temp\Temp1_Re__Aro8_blasts.zip\BD-jel_aro_uniprot.html#BL_ORD_ID:52) 5e-69

tr|A0A1L7V7G8|A0A1L7V7G8_FUSPR Related to ARO8-aromatic amino a...  [223](file:///C:\Users\Elin%20Verbrugghe\AppData\Local\Temp\Temp1_Re__Aro8_blasts.zip\BD-jel_aro_uniprot.html#BL_ORD_ID:55) 1e-67

tr|S0DIH8|S0DIH8_GIBF5 Related to ARO8-aromatic amino acid amin...  [223](file:///C:\Users\Elin%20Verbrugghe\AppData\Local\Temp\Temp1_Re__Aro8_blasts.zip\BD-jel_aro_uniprot.html#BL_ORD_ID:156) 1e-67

tr|A0A0I9YFI6|A0A0I9YFI6_GIBFU ARO8-aromatic amino acid aminotr...  [223](file:///C:\Users\Elin%20Verbrugghe\AppData\Local\Temp\Temp1_Re__Aro8_blasts.zip\BD-jel_aro_uniprot.html#BL_ORD_ID:89) 1e-67

tr|A0A319D8H9|A0A319D8H9_9EURO Aromatic aminotransferase Aro8 O...  [222](file:///C:\Users\Elin%20Verbrugghe\AppData\Local\Temp\Temp1_Re__Aro8_blasts.zip\BD-jel_aro_uniprot.html#BL_ORD_ID:79) 1e-67

tr|A0A2V5GVU1|A0A2V5GVU1_9EURO Aromatic aminotransferase Aro8 O...  [222](file:///C:\Users\Elin%20Verbrugghe\AppData\Local\Temp\Temp1_Re__Aro8_blasts.zip\BD-jel_aro_uniprot.html#BL_ORD_ID:43) 1e-67

tr|A0A2H3SUE2|A0A2H3SUE2_FUSOX Related to ARO8-aromatic amino a...  [223](file:///C:\Users\Elin%20Verbrugghe\AppData\Local\Temp\Temp1_Re__Aro8_blasts.zip\BD-jel_aro_uniprot.html#BL_ORD_ID:97) 2e-67

tr|A0A1L7S975|A0A1L7S975_FUSMA Related to ARO8-aromatic amino a...  [223](file:///C:\Users\Elin%20Verbrugghe\AppData\Local\Temp\Temp1_Re__Aro8_blasts.zip\BD-jel_aro_uniprot.html#BL_ORD_ID:84) 2e-67

tr|A1C7J1|A1C7J1_ASPCL Aromatic aminotransferase Aro8, putative...  [221](file:///C:\Users\Elin%20Verbrugghe\AppData\Local\Temp\Temp1_Re__Aro8_blasts.zip\BD-jel_aro_uniprot.html#BL_ORD_ID:34) 3e-67

tr|A0A2V5IJE2|A0A2V5IJE2_9EURO Aromatic aminotransferase Aro8 O...  [221](file:///C:\Users\Elin%20Verbrugghe\AppData\Local\Temp\Temp1_Re__Aro8_blasts.zip\BD-jel_aro_uniprot.html#BL_ORD_ID:133) 3e-67

tr|A0A319BKA8|A0A319BKA8_9EURO Aromatic aminotransferase Aro8 O...  [219](file:///C:\Users\Elin%20Verbrugghe\AppData\Local\Temp\Temp1_Re__Aro8_blasts.zip\BD-jel_aro_uniprot.html#BL_ORD_ID:119) 1e-66

tr|V5FVN7|V5FVN7_BYSSN Aromatic aminotransferase Aro8, putative...  [219](file:///C:\Users\Elin%20Verbrugghe\AppData\Local\Temp\Temp1_Re__Aro8_blasts.zip\BD-jel_aro_uniprot.html#BL_ORD_ID:56) 2e-66

tr|A0A179FF75|A0A179FF75_METCM Aromatic aminotransferase Aro8 O...  [219](file:///C:\Users\Elin%20Verbrugghe\AppData\Local\Temp\Temp1_Re__Aro8_blasts.zip\BD-jel_aro_uniprot.html#BL_ORD_ID:142) 3e-66

tr|A0A100I5Z1|A0A100I5Z1_ASPNG Aromatic aminotransferase Aro8 O...  [217](file:///C:\Users\Elin%20Verbrugghe\AppData\Local\Temp\Temp1_Re__Aro8_blasts.zip\BD-jel_aro_uniprot.html#BL_ORD_ID:47) 8e-66

tr|A0A177A0V8|A0A177A0V8_9PEZI Aromatic/aminoadipate aminotrans...  [216](file:///C:\Users\Elin%20Verbrugghe\AppData\Local\Temp\Temp1_Re__Aro8_blasts.zip\BD-jel_aro_uniprot.html#BL_ORD_ID:77) 3e-65

tr|A0A319EE16|A0A319EE16_9EURO Aromatic aminotransferase Aro8 O...  [216](file:///C:\Users\Elin%20Verbrugghe\AppData\Local\Temp\Temp1_Re__Aro8_blasts.zip\BD-jel_aro_uniprot.html#BL_ORD_ID:68) 3e-65

tr|A0A1B8GIZ3|A0A1B8GIZ3_9PEZI Aromatic/aminoadipate aminotrans...  [216](file:///C:\Users\Elin%20Verbrugghe\AppData\Local\Temp\Temp1_Re__Aro8_blasts.zip\BD-jel_aro_uniprot.html#BL_ORD_ID:153) 4e-65

tr|A0A0S3G4K4|A0A0S3G4K4_BIOOC Aromatic acid aminotransferase O...  [216](file:///C:\Users\Elin%20Verbrugghe\AppData\Local\Temp\Temp1_Re__Aro8_blasts.zip\BD-jel_aro_uniprot.html#BL_ORD_ID:71) 6e-65

tr|A0A2H3TBT2|A0A2H3TBT2_FUSOX Related to ARO8-aromatic amino a...  [214](file:///C:\Users\Elin%20Verbrugghe\AppData\Local\Temp\Temp1_Re__Aro8_blasts.zip\BD-jel_aro_uniprot.html#BL_ORD_ID:99) 8e-65

tr|A0A318Y4H9|A0A318Y4H9_9EURO Aromatic aminotransferase Aro8 O...  [214](file:///C:\Users\Elin%20Verbrugghe\AppData\Local\Temp\Temp1_Re__Aro8_blasts.zip\BD-jel_aro_uniprot.html#BL_ORD_ID:146) 1e-64

tr|A0A1S9RYU0|A0A1S9RYU0_9EURO Putative aromatic aminotransfera...  [219](file:///C:\Users\Elin%20Verbrugghe\AppData\Local\Temp\Temp1_Re__Aro8_blasts.zip\BD-jel_aro_uniprot.html#BL_ORD_ID:42) 2e-64

tr|A0A317WVZ6|A0A317WVZ6_9EURO Aromatic aminotransferase Aro8 O...  [214](file:///C:\Users\Elin%20Verbrugghe\AppData\Local\Temp\Temp1_Re__Aro8_blasts.zip\BD-jel_aro_uniprot.html#BL_ORD_ID:147) 2e-64

tr|A0A0J5PYH6|A0A0J5PYH6_ASPFM Aromatic aminotransferase Aro8 O...  [214](file:///C:\Users\Elin%20Verbrugghe\AppData\Local\Temp\Temp1_Re__Aro8_blasts.zip\BD-jel_aro_uniprot.html#BL_ORD_ID:115) 3e-64

tr|B6Q744|B6Q744_TALMQ Aromatic aminotransferase Aro8, putative...  [213](file:///C:\Users\Elin%20Verbrugghe\AppData\Local\Temp\Temp1_Re__Aro8_blasts.zip\BD-jel_aro_uniprot.html#BL_ORD_ID:160) 3e-64

tr|B8MRR4|B8MRR4_TALSN Aromatic aminotransferase Aro8, putative...  [213](file:///C:\Users\Elin%20Verbrugghe\AppData\Local\Temp\Temp1_Re__Aro8_blasts.zip\BD-jel_aro_uniprot.html#BL_ORD_ID:151) 4e-64

tr|B8MRR5|B8MRR5_TALSN Aromatic aminotransferase Aro8, putative...  [210](file:///C:\Users\Elin%20Verbrugghe\AppData\Local\Temp\Temp1_Re__Aro8_blasts.zip\BD-jel_aro_uniprot.html#BL_ORD_ID:152) 1e-63

tr|A0A179HMM5|A0A179HMM5_9HYPO Aromatic aminotransferase Aro8 O...  [213](file:///C:\Users\Elin%20Verbrugghe\AppData\Local\Temp\Temp1_Re__Aro8_blasts.zip\BD-jel_aro_uniprot.html#BL_ORD_ID:102) 1e-63

tr|G7XZZ6|G7XZZ6_ASPKW Aromatic aminotransferase Aro8 OS=Asperg...  [211](file:///C:\Users\Elin%20Verbrugghe\AppData\Local\Temp\Temp1_Re__Aro8_blasts.zip\BD-jel_aro_uniprot.html#BL_ORD_ID:67) 1e-63

tr|A0A317VFK7|A0A317VFK7_9EURO Aromatic aminotransferase Aro8 O...  [211](file:///C:\Users\Elin%20Verbrugghe\AppData\Local\Temp\Temp1_Re__Aro8_blasts.zip\BD-jel_aro_uniprot.html#BL_ORD_ID:143) 2e-63

tr|A0A2G7FM37|A0A2G7FM37_9EURO Aromatic aminotransferase Aro8 O...  [209](file:///C:\Users\Elin%20Verbrugghe\AppData\Local\Temp\Temp1_Re__Aro8_blasts.zip\BD-jel_aro_uniprot.html#BL_ORD_ID:171) 1e-62

tr|A0A0D7BLG2|A0A0D7BLG2_9AGAR Aromatic aminotransferase Aro8 O...  [207](file:///C:\Users\Elin%20Verbrugghe\AppData\Local\Temp\Temp1_Re__Aro8_blasts.zip\BD-jel_aro_uniprot.html#BL_ORD_ID:50) 3e-62

tr|A0A2D3V0C5|A0A2D3V0C5_9PEZI Related to aromatic aminotransfe...  [206](file:///C:\Users\Elin%20Verbrugghe\AppData\Local\Temp\Temp1_Re__Aro8_blasts.zip\BD-jel_aro_uniprot.html#BL_ORD_ID:62) 7e-62

tr|B8NHI9|B8NHI9_ASPFN Aromatic aminotransferase Aro8, putative...  [206](file:///C:\Users\Elin%20Verbrugghe\AppData\Local\Temp\Temp1_Re__Aro8_blasts.zip\BD-jel_aro_uniprot.html#BL_ORD_ID:154) 1e-61

tr|A0A2P2HCJ3|A0A2P2HCJ3_ASPFA Putative aromatic aminotransfera...  [206](file:///C:\Users\Elin%20Verbrugghe\AppData\Local\Temp\Temp1_Re__Aro8_blasts.zip\BD-jel_aro_uniprot.html#BL_ORD_ID:58) 1e-61

tr|A0A0E1RYA7|A0A0E1RYA7_COCIM Aromatic aminotransferase Aro8 O...  [206](file:///C:\Users\Elin%20Verbrugghe\AppData\Local\Temp\Temp1_Re__Aro8_blasts.zip\BD-jel_aro_uniprot.html#BL_ORD_ID:66) 2e-61

tr|A0A1F7ZZH4|A0A1F7ZZH4_9EURO Aromatic aminotransferase Aro8 O...  [205](file:///C:\Users\Elin%20Verbrugghe\AppData\Local\Temp\Temp1_Re__Aro8_blasts.zip\BD-jel_aro_uniprot.html#BL_ORD_ID:70) 4e-61

tr|A0A1L7WCG1|A0A1L7WCG1_9HELO Related to ARO8-aromatic amino a...  [205](file:///C:\Users\Elin%20Verbrugghe\AppData\Local\Temp\Temp1_Re__Aro8_blasts.zip\BD-jel_aro_uniprot.html#BL_ORD_ID:136) 5e-61

tr|A0A319BQ58|A0A319BQ58_9EURO Aromatic aminotransferase Aro8 (...  [205](file:///C:\Users\Elin%20Verbrugghe\AppData\Local\Temp\Temp1_Re__Aro8_blasts.zip\BD-jel_aro_uniprot.html#BL_ORD_ID:121) 5e-61

tr|A0A317VQF6|A0A317VQF6_9EURO Aromatic aminotransferase Aro8 O...  [204](file:///C:\Users\Elin%20Verbrugghe\AppData\Local\Temp\Temp1_Re__Aro8_blasts.zip\BD-jel_aro_uniprot.html#BL_ORD_ID:32) 6e-61

tr|A0A318YQ54|A0A318YQ54_9EURO Aromatic aminotransferase Aro8 O...  [204](file:///C:\Users\Elin%20Verbrugghe\AppData\Local\Temp\Temp1_Re__Aro8_blasts.zip\BD-jel_aro_uniprot.html#BL_ORD_ID:144) 7e-61

tr|A0A2W1H3P1|A0A2W1H3P1_9PLEO ARO8, Transcriptional regulator ...  [204](file:///C:\Users\Elin%20Verbrugghe\AppData\Local\Temp\Temp1_Re__Aro8_blasts.zip\BD-jel_aro_uniprot.html#BL_ORD_ID:92) 7e-61

tr|A0A146FNM7|A0A146FNM7_9EURO Aromatic aminotransferase Aro8 O...  [203](file:///C:\Users\Elin%20Verbrugghe\AppData\Local\Temp\Temp1_Re__Aro8_blasts.zip\BD-jel_aro_uniprot.html#BL_ORD_ID:148) 1e-60

tr|G7X515|G7X515_ASPKW Aromatic aminotransferase Aro8 OS=Asperg...  [203](file:///C:\Users\Elin%20Verbrugghe\AppData\Local\Temp\Temp1_Re__Aro8_blasts.zip\BD-jel_aro_uniprot.html#BL_ORD_ID:61) 1e-60

tr|A0A0F0I3R4|A0A0F0I3R4_ASPPU ARO8 like protein OS=Aspergillus...  [202](file:///C:\Users\Elin%20Verbrugghe\AppData\Local\Temp\Temp1_Re__Aro8_blasts.zip\BD-jel_aro_uniprot.html#BL_ORD_ID:65) 3e-60

tr|A0A0L1JBH0|A0A0L1JBH0_ASPNO Putative aromatic aminotransfera...  [203](file:///C:\Users\Elin%20Verbrugghe\AppData\Local\Temp\Temp1_Re__Aro8_blasts.zip\BD-jel_aro_uniprot.html#BL_ORD_ID:114) 3e-60

tr|A0A1L7TM38|A0A1L7TM38_FUSMA Related to ARO8-aromatic amino a...  [201](file:///C:\Users\Elin%20Verbrugghe\AppData\Local\Temp\Temp1_Re__Aro8_blasts.zip\BD-jel_aro_uniprot.html#BL_ORD_ID:85) 6e-60

tr|K9FG63|K9FG63_PEND2 Aromatic aminotransferase Aro8, putative...  [201](file:///C:\Users\Elin%20Verbrugghe\AppData\Local\Temp\Temp1_Re__Aro8_blasts.zip\BD-jel_aro_uniprot.html#BL_ORD_ID:169) 7e-60

tr|K9FZQ1|K9FZQ1_PEND1 Aromatic aminotransferase Aro8, putative...  [201](file:///C:\Users\Elin%20Verbrugghe\AppData\Local\Temp\Temp1_Re__Aro8_blasts.zip\BD-jel_aro_uniprot.html#BL_ORD_ID:100) 7e-60

tr|A0A179HHU7|A0A179HHU7_9HYPO Aromatic aminotransferase Aro8 O...  [200](file:///C:\Users\Elin%20Verbrugghe\AppData\Local\Temp\Temp1_Re__Aro8_blasts.zip\BD-jel_aro_uniprot.html#BL_ORD_ID:83) 2e-59

tr|A0A2H3S6R1|A0A2H3S6R1_GIBFU Related to ARO8-aromatic amino a...  [201](file:///C:\Users\Elin%20Verbrugghe\AppData\Local\Temp\Temp1_Re__Aro8_blasts.zip\BD-jel_aro_uniprot.html#BL_ORD_ID:175) 2e-59

tr|S0EDX7|S0EDX7_GIBF5 Related to ARO8-aromatic amino acid amin...  [201](file:///C:\Users\Elin%20Verbrugghe\AppData\Local\Temp\Temp1_Re__Aro8_blasts.zip\BD-jel_aro_uniprot.html#BL_ORD_ID:122) 2e-59

tr|A0A074XEX3|A0A074XEX3_AURPU Putative aromatic aminotransfera...  [200](file:///C:\Users\Elin%20Verbrugghe\AppData\Local\Temp\Temp1_Re__Aro8_blasts.zip\BD-jel_aro_uniprot.html#BL_ORD_ID:30) 2e-59

tr|A0A146F8N9|A0A146F8N9_9EURO Aromatic aminotransferase Aro8 O...  [197](file:///C:\Users\Elin%20Verbrugghe\AppData\Local\Temp\Temp1_Re__Aro8_blasts.zip\BD-jel_aro_uniprot.html#BL_ORD_ID:149) 7e-59

tr|A0A1L7W5V7|A0A1L7W5V7_FUSPR Related to ARO8-aromatic amino a...  [197](file:///C:\Users\Elin%20Verbrugghe\AppData\Local\Temp\Temp1_Re__Aro8_blasts.zip\BD-jel_aro_uniprot.html#BL_ORD_ID:53) 1e-58

tr|A0A2W1HDD9|A0A2W1HDD9_9PLEO ARO8, Transcriptional regulator ...  [204](file:///C:\Users\Elin%20Verbrugghe\AppData\Local\Temp\Temp1_Re__Aro8_blasts.zip\BD-jel_aro_uniprot.html#BL_ORD_ID:113) 2e-58

tr|A0A1T3CCB0|A0A1T3CCB0_9HYPO ARO8, Transcriptional regulator ...  [197](file:///C:\Users\Elin%20Verbrugghe\AppData\Local\Temp\Temp1_Re__Aro8_blasts.zip\BD-jel_aro_uniprot.html#BL_ORD_ID:145) 3e-58

tr|A0A319D396|A0A319D396_9EURO Aromatic aminotransferase Aro8 O...  [194](file:///C:\Users\Elin%20Verbrugghe\AppData\Local\Temp\Temp1_Re__Aro8_blasts.zip\BD-jel_aro_uniprot.html#BL_ORD_ID:130) 2e-57

tr|A0A1B2JEQ2|A0A1B2JEQ2_PICPA BA75_04174T0 OS=Komagataella pas...  [193](file:///C:\Users\Elin%20Verbrugghe\AppData\Local\Temp\Temp1_Re__Aro8_blasts.zip\BD-jel_aro_uniprot.html#BL_ORD_ID:131) 3e-57

tr|A0A0J0XSH6|A0A0J0XSH6_9TREE Aromatic aminotransferase Aro8 O...  [194](file:///C:\Users\Elin%20Verbrugghe\AppData\Local\Temp\Temp1_Re__Aro8_blasts.zip\BD-jel_aro_uniprot.html#BL_ORD_ID:88) 3e-57

tr|A0A132B9F5|A0A132B9F5_9HELO Aromatic aminotransferase Aro8 O...  [194](file:///C:\Users\Elin%20Verbrugghe\AppData\Local\Temp\Temp1_Re__Aro8_blasts.zip\BD-jel_aro_uniprot.html#BL_ORD_ID:167) 4e-57

tr|A0A1S7HP10|A0A1S7HP10_9SACH ARO8 (YGL202W) OS=Zygosaccharomy...  [191](file:///C:\Users\Elin%20Verbrugghe\AppData\Local\Temp\Temp1_Re__Aro8_blasts.zip\BD-jel_aro_uniprot.html#BL_ORD_ID:172) 2e-56

tr|F2QQ55|F2QQ55_KOMPC Aromatic/aminoadipate aminotransferase 1...  [191](file:///C:\Users\Elin%20Verbrugghe\AppData\Local\Temp\Temp1_Re__Aro8_blasts.zip\BD-jel_aro_uniprot.html#BL_ORD_ID:35) 3e-56

tr|A0A2V5HT04|A0A2V5HT04_9EURO Aromatic aminotransferase Aro8 O...  [191](file:///C:\Users\Elin%20Verbrugghe\AppData\Local\Temp\Temp1_Re__Aro8_blasts.zip\BD-jel_aro_uniprot.html#BL_ORD_ID:155) 4e-56

tr|A0A2D3VAY5|A0A2D3VAY5_9PEZI Related to ARO8-aromatic amino a...  [191](file:///C:\Users\Elin%20Verbrugghe\AppData\Local\Temp\Temp1_Re__Aro8_blasts.zip\BD-jel_aro_uniprot.html#BL_ORD_ID:82) 5e-56

tr|A0A1E1MDU4|A0A1E1MDU4_RHYSE Related to ARO8-aromatic amino a...  [190](file:///C:\Users\Elin%20Verbrugghe\AppData\Local\Temp\Temp1_Re__Aro8_blasts.zip\BD-jel_aro_uniprot.html#BL_ORD_ID:116) 9e-56

tr|A0A1E1KD92|A0A1E1KD92_9HELO Related to ARO8-aromatic amino a...  [190](file:///C:\Users\Elin%20Verbrugghe\AppData\Local\Temp\Temp1_Re__Aro8_blasts.zip\BD-jel_aro_uniprot.html#BL_ORD_ID:132) 1e-55

tr|A0A1E1KXI8|A0A1E1KXI8_9HELO Related to ARO8-aromatic amino a...  [190](file:///C:\Users\Elin%20Verbrugghe\AppData\Local\Temp\Temp1_Re__Aro8_blasts.zip\BD-jel_aro_uniprot.html#BL_ORD_ID:41) 1e-55

tr|A0A1S7HCZ5|A0A1S7HCZ5_9SACH ARO8 (YGL202W) OS=Zygosaccharomy...  [189](file:///C:\Users\Elin%20Verbrugghe\AppData\Local\Temp\Temp1_Re__Aro8_blasts.zip\BD-jel_aro_uniprot.html#BL_ORD_ID:174) 2e-55

tr|A0A0S3G4L3|A0A0S3G4L3_BIOOC Aromatic acid aminotransferase O...  [189](file:///C:\Users\Elin%20Verbrugghe\AppData\Local\Temp\Temp1_Re__Aro8_blasts.zip\BD-jel_aro_uniprot.html#BL_ORD_ID:36) 2e-55

tr|V5IRM1|V5IRM1_NEUCR Aromatic aminotransferase Aro8, variant ...  [188](file:///C:\Users\Elin%20Verbrugghe\AppData\Local\Temp\Temp1_Re__Aro8_blasts.zip\BD-jel_aro_uniprot.html#BL_ORD_ID:93) 9e-55

tr|A0A2H3TRL5|A0A2H3TRL5_FUSOX Related to aromatic aminotransfe...  [186](file:///C:\Users\Elin%20Verbrugghe\AppData\Local\Temp\Temp1_Re__Aro8_blasts.zip\BD-jel_aro_uniprot.html#BL_ORD_ID:98) 3e-54

tr|A0A1D8PG20|A0A1D8PG20_CANAL Bifunctional 2-aminoadipate tran...  [185](file:///C:\Users\Elin%20Verbrugghe\AppData\Local\Temp\Temp1_Re__Aro8_blasts.zip\BD-jel_aro_uniprot.html#BL_ORD_ID:2) 3e-54

tr|A0A1X7QY12|A0A1X7QY12_9SACH Similar to Saccharomyces cerevis...  [185](file:///C:\Users\Elin%20Verbrugghe\AppData\Local\Temp\Temp1_Re__Aro8_blasts.zip\BD-jel_aro_uniprot.html#BL_ORD_ID:137) 4e-54

tr|A0A1Y2EGT2|A0A1Y2EGT2_9BASI Aromatic aminotransferase Aro8 O...  [185](file:///C:\Users\Elin%20Verbrugghe\AppData\Local\Temp\Temp1_Re__Aro8_blasts.zip\BD-jel_aro_uniprot.html#BL_ORD_ID:157) 5e-54

tr|H0GUJ4|H0GUJ4_SACCK Aro8p OS=Saccharomyces cerevisiae x Sacc...  [183](file:///C:\Users\Elin%20Verbrugghe\AppData\Local\Temp\Temp1_Re__Aro8_blasts.zip\BD-jel_aro_uniprot.html#BL_ORD_ID:48) 2e-53

tr|W0T517|W0T517_KLUMD Aromatic amino acid aminotransferase 1 O...  [182](file:///C:\Users\Elin%20Verbrugghe\AppData\Local\Temp\Temp1_Re__Aro8_blasts.zip\BD-jel_aro_uniprot.html#BL_ORD_ID:59) 4e-53

tr|A0A0H5BY90|A0A0H5BY90_CYBJA ARO8 protein OS=Cyberlindnera ja...  [181](file:///C:\Users\Elin%20Verbrugghe\AppData\Local\Temp\Temp1_Re__Aro8_blasts.zip\BD-jel_aro_uniprot.html#BL_ORD_ID:75) 6e-53

tr|A0A0B6VRD9|A0A0B6VRD9_ZYGRO Aromatic aminotransferase OS=Zyg...  [181](file:///C:\Users\Elin%20Verbrugghe\AppData\Local\Temp\Temp1_Re__Aro8_blasts.zip\BD-jel_aro_uniprot.html#BL_ORD_ID:170) 8e-53
