# Supplementary material for: Growth Regulation in Amphibian Pathogenic Chytrid Fungi by the Quorum Sensing Metabolite Tryptophol
Source: Front Microbiol. 2019 Jan 8;9:3277. doi: 10.3389/fmicb.2018.03277 (PMC6331427; doi:10.3389/fmicb.2018.03277)
Supplement: Supplementary file 2 [file Table_2.DOCX]

**Supplementary table 2: Overview of the statistical tests performed.**

| **Figure** | **Comparisons** | **Normally distributed** | **Equal variances** | **Statistical test** | **P-value for significance** |
| --- | --- | --- | --- | --- | --- |
| 1A | TGhL vs 20%  TGhL vs 40%  TGhL vs 60%  TGhL vs 80%  TGhL vs 100 % | no | no | Kruskal-wallis followed by a Bonferroni- corrected Mann-Whitney U test | p < 0.01 (0.05/5) |
|  | H2O vs CM | no | no | Kruskal-wallis | p < 0.05 |
| 1B | TGhL vs 40% H2O  TGhL vs 40% CM1  TGhL vs 40% CM2  TGhL vs 40% CM3  TGhL vs 40% CM4  TGhL vs 40% CM5 | yes | yes | One-way ANOVA with Bonferroni as post hoc | p < 0.05 |
| 1C | 40% CM vs TGhL  40% CM vs 40% H2O  40% CM vs 500 Da  40% CM vs 1000 Da | yes | yes | One-way ANOVA with Bonferroni as post hoc | p < 0.05 |
| 2B | control vs 10 µM  control vs 25 µM  control vs 50 µM  control vs 100 µM  control vs 250 µM  control vs 500 µM | no | no | Kruskal-wallis followed by a Bonferroni- corrected Mann-Whitney U test | p < 0.0083 (0.05/6) |
| 5A | control vs 0.001 µM  control vs 0.01 µM  control vs 0.1 µM  control vs 1 µM | no | no | Kruskal-wallis followed by a Bonferroni- corrected Mann-Whitney U test | p < 0.0125 (0.05/4) |
| 5B | spores low vs spores high | no | no | Kruskal-wallis | p < 0.05 |
| 5C | spores low vs *Bd*-infected tissue | no | no | Kruskal-wallis | p < 0.05 |
