# Supplementary material for: Growth Regulation in Amphibian Pathogenic Chytrid Fungi by the Quorum Sensing Metabolite Tryptophol
Source: Front Microbiol. 2019 Jan 8;9:3277. doi: 10.3389/fmicb.2018.03277 (PMC6331427; doi:10.3389/fmicb.2018.03277)
Supplement: Supplementary file 3 [file Data_Sheet_1.PDF]

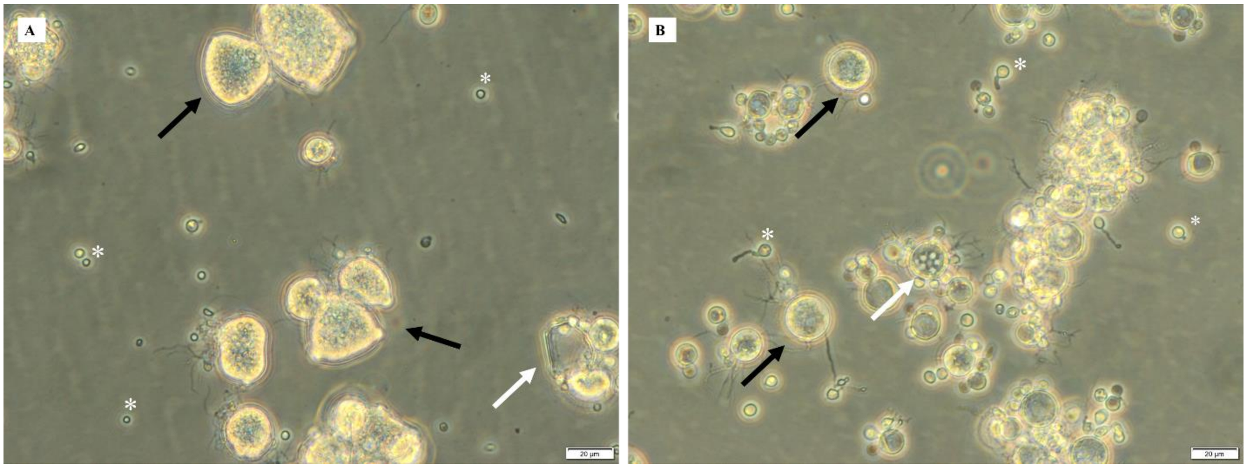

Supplementary Figure 1: ***In vitro* culture of chytrid fungi. (A)** Morphology of *Bd* in TGhL broth at 20°C, showing abundant mature zoosporangia (black arrow) containing zoospores, empty discharged sporangia (white arrow), and motile zoospores (white asterisk). **(B)** Morphology of *Bsal* in TGhL broth at 15°C, characterized by predominant monocentric thalli (black arrow), sporulating zoosporangia (white arrow) and zoospore cysts with germ tubes (white asterisk).
