# Supplementary material for: Growth Regulation in Amphibian Pathogenic Chytrid Fungi by the Quorum Sensing Metabolite Tryptophol
Source: Front Microbiol. 2019 Jan 8;9:3277. doi: 10.3389/fmicb.2018.03277 (PMC6331427; doi:10.3389/fmicb.2018.03277)
Supplement: Supplementary file 4 [file Data_Sheet_2.PDF]

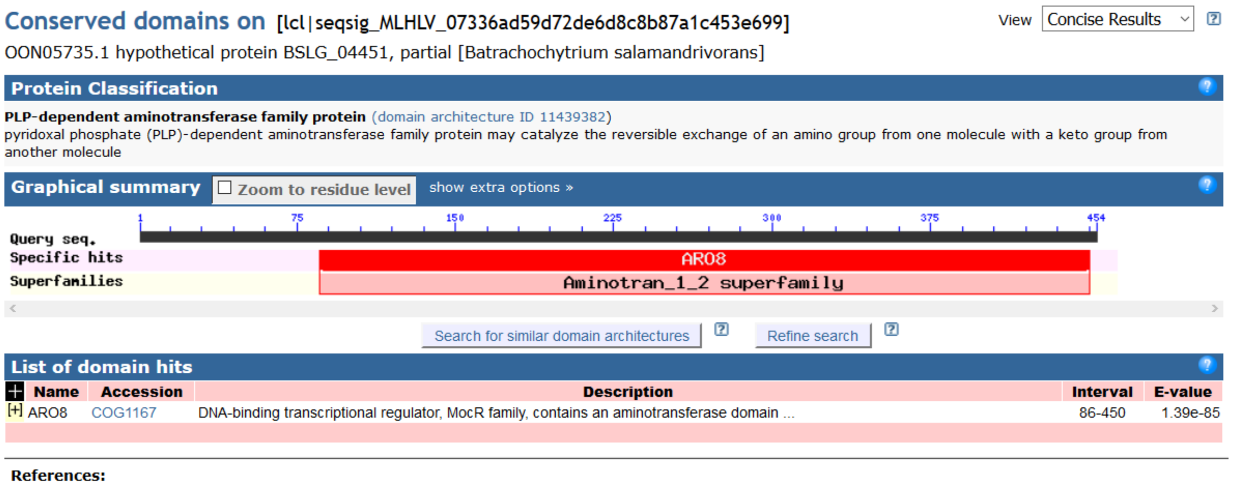

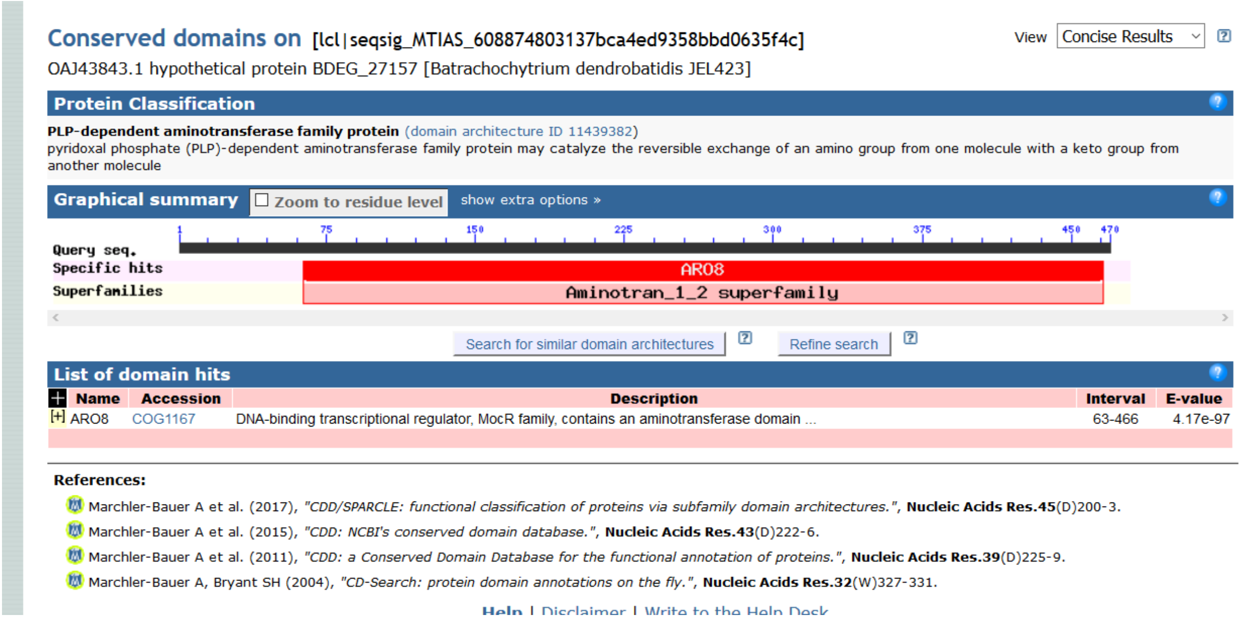

Supplementary Figure 2: Conserved domains search. Using the NCBI's Conserved Domains database, a conserved domain search of BSLG_04451 (OON05735.1) and BDEG_27157 (AJ43843.1) was performed against ARO8 of *Saccharomyces cerevisiae* (BioProject: PRJNA128, Accession: NP_011313).
