# Supplementary material for: Growth Regulation in Amphibian Pathogenic Chytrid Fungi by the Quorum Sensing Metabolite Tryptophol
Source: Front Microbiol. 2019 Jan 8;9:3277. doi: 10.3389/fmicb.2018.03277 (PMC6331427; doi:10.3389/fmicb.2018.03277)
Supplement: Supplementary file 5 [file Data_Sheet_3.PDF]

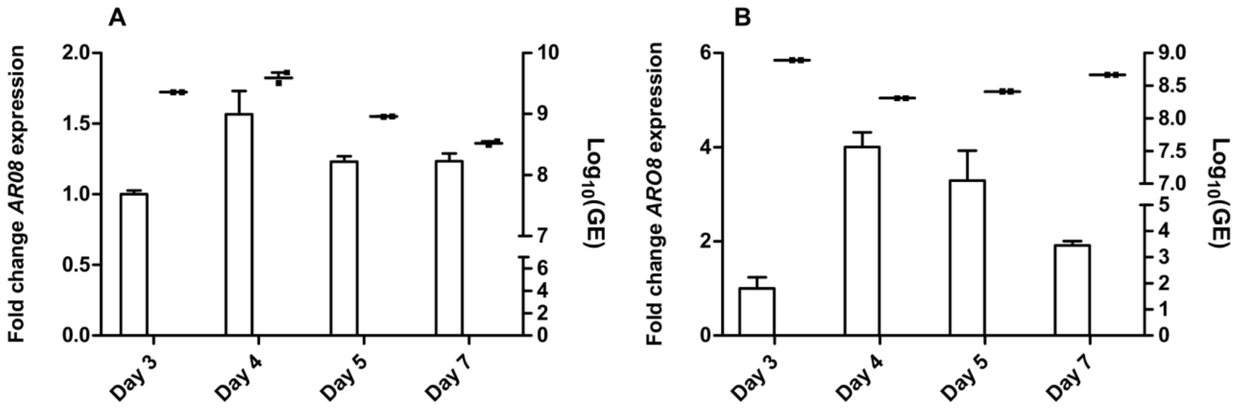

Supplementary figure 3: Correlation *Bd* and *Bsal* growth to *ARO8* expression. The data (histograms) show the normalized *ARO8* gene expression at different days during growth of a **(A)** *Bd* and **(B)** *Bsal* culture, relative to the growth at day 3 which is considered 1. The Log_{10} of the GE numbers is depicted by black dots. Results represent the mean + SEM.
